# Widespread purifying selection on RNA structure in mammals

**DOI:** 10.1093/nar/gkt596

**Published:** 2013-07-11

**Authors:** Martin A. Smith, Tanja Gesell, Peter F. Stadler, John S. Mattick

**Affiliations:** ^1^RNA Biology and Plasticity Laboratory, Garvan Institute of Medical Research, 384 Victoria Street, Darlinghurst, Sydney, NSW 2010 Australia, ^2^Genomics and Computational Biology Division, Institute for Molecular Bioscience, 306 Carmody Rd, University of Queensland, Brisbane, 4067 Australia, ^3^Department of Structural and Computational Biology; and Center for Integrative Bioinformatics Vienna (CIBIV), Max F. Perutz Laboratories (MFPL), University of Vienna, Medical University of Vienna, Dr. Bohr-Gasse 9, A-1030 Vienna, Austria, ^4^Bioinformatics Group, Department of Computer Science; and Interdisciplinary Center for Bioinformatics, University of Leipzig, Härtelstrasse 16–18, D-04107 Leipzig, Germany, ^5^Max Planck Institute for Mathematics in the Sciences, Inselstraße 22, D-04103 Leipzig, Germany, ^6^Center for Non-coding RNA in Technology and Health, Department of Basic Veterinary and Animal Sciences, Faculty of Life Sciences University of Copenhagen, Grønnegårdsvej 3, 1870 Frederiksberg C Denmark, ^7^Santa Fe Institute, 1399 Hyde Park Rd, Santa Fe, NM 87501, USA and ^8 ^St Vincent’s Clinical School, University of New South Wales, Level 5, de Lacy, Victoria St, St Vincent's Hospital, Sydney, NSW 2010 Australia

## Abstract

Evolutionarily conserved RNA secondary structures are a robust indicator of purifying selection and, consequently, molecular function. Evaluating their genome-wide occurrence through comparative genomics has consistently been plagued by high false-positive rates and divergent predictions. We present a novel benchmarking pipeline aimed at calibrating the precision of genome-wide scans for consensus RNA structure prediction. The benchmarking data obtained from two refined structure prediction algorithms, RNAz and SISSIz, were then analyzed to fine-tune the parameters of an optimized workflow for genomic sliding window screens. When applied to consistency-based multiple genome alignments of 35 mammals, our approach confidently identifies >4 million evolutionarily constrained RNA structures using a conservative sensitivity threshold that entails historically low false discovery rates for such analyses (5–22%). These predictions comprise 13.6% of the human genome, 88% of which fall outside any known sequence-constrained element, suggesting that a large proportion of the mammalian genome is functional. As an example, our findings identify both known and novel conserved RNA structure motifs in the long noncoding RNA *MALAT1*. This study provides an extensive set of functional transcriptomic annotations that will assist researchers in uncovering the precise mechanisms underlying the developmental ontologies of higher eukaryotes.

## INTRODUCTION

The majority of the human genome is dynamically transcribed into RNA, most of which does not code for proteins (1–4). The once common presumption that most non–protein-coding sequences are nonfunctional for the organism is being adjusted to the increasing evidence that noncoding RNAs (ncRNAs) represent a previously unappreciated layer of gene expression essential for the epigenetic regulation of differentiation and development (5–8). Yet despite an exponential accumulation of transcriptomic data and the recent dissemination of genome-wide data from the ENCODE consortium (9), limited functional data have fuelled discourse on the amount of functionally pertinent genomic sequence in higher eukaryotes (1,10–12). What is incontrovertible, however, is that evolutionary conservation of structural components over an adequate evolutionary distance is a direct property of purifying (negative) selection and, consequently, a sufficient indicator of biological function. The majority of studies investigating the prevalence of purifying selection in mammalian genomes are predicated on measuring nucleotide substitution rates, which are then rated against a statistical threshold trained from a set of genomic loci arguably qualified as neutrally evolving (13,14).

Conversely, lack of conservation does not impute lack of function, as variation underlies natural selection. Given that the molecular function of ncRNA may at least be partially conveyed through secondary or tertiary structures, mining evolutionary data for evidence of such features promises to increase the resolution of functional genomic annotations. In this case, higher substitution rates observed in evolutionary data can nonetheless be informative of conserved base pairings in higher-order structural interactions. When RNAs function through their 2D or 3D structural conformations, mutations are often tolerated provided that they maintain complementary base pairing—an occurrence termed covariation, which is a *bona fide* indication of evolutionary selection on RNA structure (15). The mutational flexibility of ncRNA allows for faster evolutionary substitution rates than proteins, entailing discrete patterns of mutation that are sufficient to predict function (16).

Current technology renders 3D structural prediction unfeasible for high-throughput comparative transcriptomics. However, computational analyses predicated on secondary structures—canonical Watson–Crick base pairings that form stable helices—are well established and tractable for large genomes. Indeed, there is already strong evidence that RNA is subject to evolutionary preservation of secondary structure conformations in mammalian species.

A handful of RNA secondary structure prediction tools of various flavors have already been applied to pan-genomic screens. The *modus operandi* of these algorithms usually involves the sampling of genomic multiple sequence alignments via consecutive overlapping windows of fixed size (the ‘sliding window’ approach). Past studies have used EvoFold (17), CMFinder (18), RNAz (19) and AlifoldZ (20) on multiple genome alignments associated to ENCODE pilot project loci (21).

EvoFold implements phylogenetic stochastic context-free grammars to evaluate how well nucleotide substitutions correlate to sampled secondary structure topologies (17). Its predictions are strongly biased toward conserved genomic sequences and AU-rich alignments (20).

RNAz uses a support vector machine (SVM) trained on evolutionary conservation and thermodynamic stability scores derived from known structured RNAs and randomized background to emit consensus structure predictions (22). It has been reported that RNAz predicts secondary structures with a high false-positive rate and a bias toward strong GC content, although its speed may supersedes these caveats.

The AlifoldZ algorithm uses the consensus 2D structure prediction algorithm RNAalifold to compare the consensus RNA secondary structure score of a native alignment against the distribution of scores derived from shuffled alignments (23–25). The algorithm also suffers from slow runtimes and a high false-positive rate ensued by background modeling through shuffling of individual columns of the queried multiple sequence alignment. This approach produces a background model without consideration of dinucleotide frequencies, which have been shown to increase the specificity of RNA structure prediction through better representation of the thermodynamic free energies of base-pair stacking (26,27).

The SISSI algorithm offers a solution to this problem by producing dinucleotide-controlled, simulated alignments through modeling of site-specific nucleotide interactions in function of the branch lengths from a phylogenetic tree inferred from the original alignment (28). The entailing simulated alignments, which maintain the global sequence characteristics of the original alignment, can then be used as a background distribution for scoring RNA secondary structure conservation with RNAalifold, as implemented in SISSIz (29). This algorithm was also used to improve the specificity of a revised version of RNAz (30). Both SISSIz and the revised version of RNAz have yet to be applied to genome-wide screens.

In contrast to the above-mentioned programs, CMFinder essentially realigns the input alignment via an iterative statistical method for optimizing a multiple alignment of RNA structures (31). CMFinder’s robustness comes at the expense of computational complexity, a high false-positive rate (∼50%), and the need to manually adjust certain user-defined parameters. In addition to these conventional methods, there are other algorithms that calculate common RNA secondary structures to a set of orthologous sequences [reviewed in (32–36)].

A prevalent shortcoming of Evolutionarily Conserved Structure (ECS) prediction algorithms and methodologies used for genomic screens is the lack of a diverse set of true positives presenting the evolutionary signatures of selection for RNA structure. With the exception of the output from the above-mentioned studies, which seldom overlap, there are few known examples of mammalian ECSs that cannot be detected through purely sequence-based approaches (e.g. rRNAs). The prediction algorithms are usually trained and benchmarked on refined structural alignments of individual molecules, namely the RNA FAMily database (RFAM) and BRAliBASE (37–39). These offer ideal conditions to make predictions yet do not represent actual experimental conditions; published results are chiefly derived from fragmented sequence-based genome alignments generated by speedy heuristics, such as the TBA/Multiz data sets from the UCSC genome browser (40,41). Furthermore, ECS prediction endeavors often use a sliding-window approach, where the boundaries of potential structural elements are not guaranteed to coincide with the sampled alignment (34). There is hence need for an objective measure of the efficiency of ECS prediction algorithms in line with implemented high-throughput methodologies to improve the quality and confidence of resulting experiential predictions. This necessity is of particular significance now that sequencing technologies are more available and will inevitably generate considerable evolutionary information—an outcome that will contrast greater alignment depths with shorter syntenic alignment blocks to accommodate the broader diversity of genome organizations.

Here, we investigate the prevalence of potentially functional sequences in the expanses of mammalian genomes with undetermined function by investigating evolutionary patterns of genetic variation consistent with the formation of RNA secondary structures. Through an original benchmarking pipeline that reproduces the experimental conditions of genome-wide sliding window methodologies, we improve the confidence and resolution of consensus-based ECS predictions with SISSIz and RNAz by creating tailor-made data sets of known structured RNAs and negative controls. Optimal prediction parameters are then extended to a high-throughput genomic screen via a massively parallel composite algorithm, which faithfully detects evolutionary patterns of structural conservation. This is achieved with little sequence composition bias for any sampled alignment, thus overcoming caveats arising from regions of weaker sequence conservation. Our approach exposes millions of novel genomic loci presenting strong evidence for purifying selection at the level of RNA secondary structure, while maintaining high specificity and reasonable computational complexity.

## MATERIALS AND METHODS

### Data generation and benchmarking pipeline

**Figure 1.**
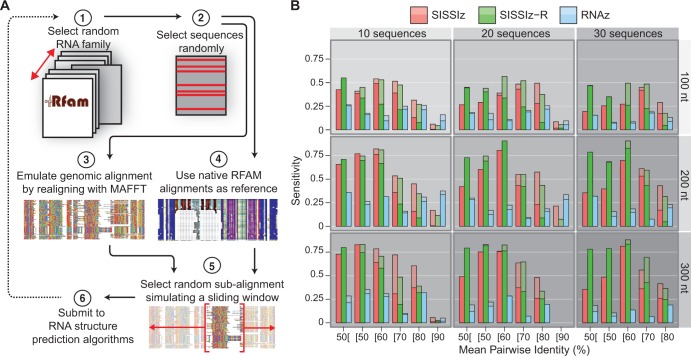
Benchmarking the sensitivity of sliding-window RNA structure prediction. (**A**) Benchmarking pipeline for simulating the experimental conditions of sliding-window methodologies using known RNA structure alignments. (**B**) The relative sensitivities of conserved RNA secondary structure prediction algorithms are plotted for randomly sampled, native RFAM subalignments in function of the amount of sequences, window length and MPI. Opaque bars represent high-confidence predictions (RNAz probability ≥ 0.9, SISSIz Z-score ≤ −4), while translucent bars represent lower-confidence predictions (RNAz probability ≥ 0.5, SISSIz Z-score ≤ −2). Each bar represents the outcome of 200 sampled alignments with RNAz version 2.0 (using options ‘-f–d–l’), SISSIz using default parameters and SISSIz with RIBOSUM scoring (option ‘-j’) for all indicated window sizes, sequence depths and MPI ranges. The latter are indicated by their bounded values on the x-axis.

The full alignments of 89 structural RNA families containing at least one mammalian sequence (excluding miRNAs, tRNAs and most snoRNAs) were downloaded from RFAM release 10.0 (ftp://ftp.sanger.ac.uk/pub/databases/Rfam/10.0/; Supplementary Data S1). Our algorithm first selects one of the alignment files (converted from stockholm to fasta format) at random, from which 10, 20 and 30 sequences were randomly extracted. At this stage, an optional realignment step can be performed to emulate the experimental conditions encountered in genome-wide screens. The MAFFT-GINSI (42) alignment program that uses fast Fourier transforms and iterative refinement was used as it is renowned to perform well on structured RNA alignments (43,44). It also has the advantage of being fast enough for realigning alignment fragments at the genome scale. Once a subset of sequences is selected, empty columns are removed and then a subalignment—or window—of specified length is chosen randomly within the full alignment (Supplementary Data S2–S3), thus mimicking the sampling procedure in a genome-wide screen using sliding windows. A schematic of this pipeline is illustrated in Figure 1A. Sensitivity and specificity are measured as true positives/(true positives + false negatives) and true negatives/(true negatives + false positives), respectively.

The mean pairwise identity (MPI—defined as the average amount of identities divided by the length of the shortest sequence for all sequence pairs), the standard deviation of the MPI, the normalized Shannon entropy (45), GC content and gap content are calculated for all sampled alignments. Next, the RFAM structure annotation is compared with the RNAalifold consensus structure prediction (with default scoring and RIBOSUM scoring metric variants), using ‘RNAdistance -DP’ from the Vienna RNA package version 1.8.5 (http://www.tbi.univie.ac.at/RNA/) (46). The alignments are then scored with SISSIz version 2.0 (29) (updated binaries can be downloaded from the Supplementary Information) using default parameters (‘-j’ option for RIBOSUM scoring) and with RNAz version 2.0 (30) using the following parameters: ‘-f -d’ for MAFFT alignments; ‘-f -t -l’ for native RFAM structural alignments. Finally, the subalignments are saved and the process is reiterated until a sufficient amount of subalignments is obtained for all user-defined MPI ranges. The algorithm is implemented in JAVA and can be downloaded from a link in the Supplementary Information.

### Multiple genome alignments

Enredo-Pecan-Ortheus (EPO) consistency-based multiple alignments of 35 eutherian mammals for human genome assembly GRCh37 were downloaded from ENSEMBL-compara release 65 (ftp://ftp.ensembl.org/pub/release-65/emf/ensembl-compara/epo_35_eutherian/) and converted from EMF to MAF format. Segmental duplications were removed from each alignment block, when present, to ensure that no more than one sequence from each species was surveyed.

### Generating true-negative controls

Previous studies have evaluated specificity through negative controls by using genomic alignments from intergenic regions (29) or by permuting the positions of individual columns or bases in the alignments (17–20). While the latter falsely increases specificity through disruption of dinucleotide composition, the former assumes these regions are not transcribed, ergo are unlikely to undergo purifying selection for higher-order structure. We tested several different strategies for generating true negatives, including (i) employing the independent program MULTIPERM to shuffle each sampled alignment window before making predictions (47); (ii) combining two shuffling algorithms, i.e. using the simulation algorithm (SISSI) of SISSIz on the entire syntenic alignment block, then MULTIPERM on individually sampled windows. The last approach ensures that any algorithm-specific biases for null model generation are reduced through the combined action of both tools. It also avoids using the same null model to generate the simulated alignment and the ECS prediction because different alignments are used to train the model. All shuffled alignments were performed for human chromosome 10 (an average-sized chromosome) on EPO 35-way alignments (as described above) using a window size of 200 nt, a 100-nt step, and the same quality control filters as described below.

### Hybrid algorithm for optimized ECS prediction

Our algorithm first reads a MAF (Multiple Alignment Format) file that can optionally be stripped of indels and realigned with MAFFT-GINSI (42). Input alignments are then split into sliding windows of 200 nt that overlap by 100 nt. Identical sequences are removed just as those with ≥50% gaps or ambiguous characters. Alignments with three or more sequences, including human, are then reverse-complemented and their sequence characteristics are calculated (MPI, standard deviation of the MPI, normalized Shannon entropy, GC content, gap content). The underlying ECS prediction algorithm selection parameters are as follows: RNAz is used when the MPI is >85%; SISSIz is used when MPI is between 60 and 85% and when GC content is <70%; otherwise, SISSIz with RIBOSUM scoring is used. SISSIz with RIBOSUM is also used when RNAalifold fails to predict a consensus structure or when SISSIz’s background distribution presents a standard deviation <0.5, which can produce extreme Z-scores (Supplementary Figure S5). Based on preliminary data, we selected thresholds of −2.7, −2.2 and 0.32 for SISSIz, SISSIz+RIBOSUM and RNAz predictions, respectively, for reporting ECS predictions.

The program outputs a six-field browser extensible data file with the sequence characteristics in the name field, and the RNAz or SISSIz score in the score field, while filtered alignments corresponding to hits presenting scores >99% specificity thresholds are saved (Supplementary Figure S6). The user-friendly pipeline is implemented in JAVA, is compatible with high-performance computing infrastructures and can be accessed through the Supplementary Information.

### Structural congruence analysis pipeline

We define structural congruence as how well an individual sequence complies with the evolutionarily conserved consensus structure. The structural congruence of all ECS predictions was evaluated 2-fold: (i) by measuring the relative difference between both base pairing probabilities as calculated with McCaskill’s partition function algorithm, and (ii) by comparing the thermodynamic stability of both folds, a metric akin to RNAz’s structure conservation index. The RNAalifold consensus dot-bracket annotation is extracted for all alignments that generate a high-confidence prediction (‘RNAalifold -r’ is used for hits generated with the RIBOSUM variant of SISSIz). The reference sequence is then processed to remove gaps, while the corresponding positions in the consensus structure are removed, in addition to any unpaired positions that may arise in the consensus structure. Next, the reference sequence is submitted to partition function folding as implemented in ‘RNAfold -p’ from the Vienna RNA package version 1.8.5 [(46); http://www.tbi.univie.ac.at/RNA/] from which the MFE structure annotation is extracted. The resulting base pairing probability matrix is then used to calculate the relative difference in folding probabilities for both structure annotations in the following manner:

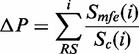

with

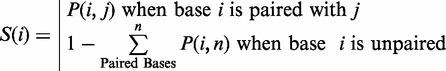

where Δ*P* is the ratio of base-pairing probabilities, *RS* is the reference sequence, *S_mfe_* and *S*_c_ are the minimum free energy and consensus structure annotations, respectively. The relative difference in free energies is also measured as the ratio of the constrained structure’s free energy normalized to the unrestricted minimum free energy. A reference sequence (human in this case) is qualified as structurally congruent to an alignment’s consensus structure when both of these ratios are >0.75 and when either one is >0.9. The pipeline is implemented in JAVA and is available for download in the Supplementary Information.

## RESULTS

### Performance evaluation of ECS prediction algorithms

We tested the performance of two recent ECS prediction algorithms suited for genome-wide screens of conserved RNA secondary structure from multiple sequence alignments, namely SISSIz and RNAz 2.0 (hereafter referred to as RNAz). Both SISSIz and RNAz evaluate the likelihood of evolutionary selection on higher-order structure by comparing structure conservation and sequence conservation within any given multiple alignment sample. In other words, these tools test whether the consensus RNA secondary structure is more divergent than expected from the observed sequences. RNAz 2.0 and SISSIz emit consensus structure predictions with greater specificity than the other tools mentioned above and have not yet been applied to genome-wide screen in mammals. We revised SISSIz to incorporate the latest RNAalifold scoring metrics, which enable the use of RIBOSUM substitution matrices that improve consensus RNA secondary structure significantly (23). By comparing the patterns of base substitutions in sampled alignments to the substitution patterns observed in the evolutionary history of ribosomal RNAs, a RIBOSUM-empowered version of SISSIz should enable stronger discrimination between genomic regions harboring higher-order structures and those that do not.

Currently, the most extensive database of structured RNAs is RFAM (37), which contains homologous, hand curated RNA families with well-characterized secondary structural components. The inherent alignments are of structural nature, where homologous helical regions (i.e. stems) and unpaired regions are aligned together through manual intervention or from the output of sophisticated statistical models (48). RFAM alignments provide an ideal data set of positive controls for RNA structure prediction algorithms that consider consistent (e.g. G:C → G:U) and compensatory (e.g. G:C → A:U) base substitutions that preserve base pairings in helical RNA structures. Consequently, we tested the performance of SISSIz and RNAz on 89 full RFAM alignments with at least one mammalian representative, totaling 186 662 curated RNA structures (see ‘Materials and Methods’ section). On average, each selected RNA family contains a median value of 154 sequences with 59.4% of them being unique (Supplementary Figure S3), thus providing a substantially more diverse data set than BRAliBASE (39).

Because the most computationally tractable approach to genome-wide scans is to sample multiple genomic alignments iteratively via sliding windows of fixed length and overlap, it is not unlikely that the boundaries of natural RNA structures occur outside a given window position. We developed a benchmarking pipeline that reproduces this limitation by selecting random subalignments from the RFAM data to evaluate the sensitivity of ECS prediction algorithms in experimental conditions (Figure 1A). To measure the impact of sequence conservation on ECS prediction, we iterated the process for different MPI values to generate multiple ‘bins’ of varying sequence conservation. Figure 1 illustrates the impact of the amount of surveyed sequences, the window length and sequence similarity on the sensitivity (as defined in ‘Materials and Methods’ section) of RNAz, SISSIz and SISSIz using RIBOSUM scoring. Using a statistical cutoff of 4 standard deviations from the mean of each sampled subalignment’s background distribution (P < 1.5×10^−^^5^), SISSIz produces on average more than twice as many predictions than high-confidence RNAz predictions (Figure 1, Table 1). This difference is most remarkable when alignments present MPI values between 50 and 80%, while RNAz performs best on alignments with higher sequence conservation for the applied confidence thresholds. The RIBOSUM-enabled version of SISSIz performs particularly well with alignments of low sequence homology (<60%), which are referred to as the ‘twilight zone’ of multiple sequence alignments where sequence alignment algorithms perform poorly. Genomic regions presenting sequence conservation values in this range are regularly classified as not being subject to negative selection, suggesting that SISSIz with RIBOSUM scoring has the potential to uncover putative functional genomic regions that have previously been ignored.

**Table 1.** Accuracy of SISSIz and RNAz on RFAM sub-alignments

Alignment source	Algorithm	Sensitivity^a^	Specificity^a^^,^^b^	
			*SISSIz-s*	*Multiperm*
**Native RFAM**	RNAz	17.7	99.8	100
	SISSIz	37.8	99.0	92.3
	SISSIz + RIBOSUM	40.9	99.2	90.8
**Emulated genomic^c^**	RNAz	16.1	99.7	99.8
	SISSIz	30.3	99.0	93.2
	SISSIz + RIBOSUM	34.3	99.6	93.0

^a^Using a confidence threshold half way between those reported in Figure 1 (Z-score ≤ −3 for SISSIz; *P* ≥ 0.75 for RNAz).

^b^From the associated dinucleotide-controlled shuffled alignments.

^c^Selected RFAM sequences are de-gapped and realigned with MAFFT-GINSI (42). RNAz predictions for alignments >300 nt were not calculated.

Values reflect the cumulative specificity for 10 200 and 6800 alignments of 10, 20 and 30 sequences with window lengths of 100, 200, 300 for SISSIz; 100 and 200 for RNAz, respectively. RNAz scoring for native RFAM alignments used the ‘−l’ option for structure alignments.

**Figure 2.**
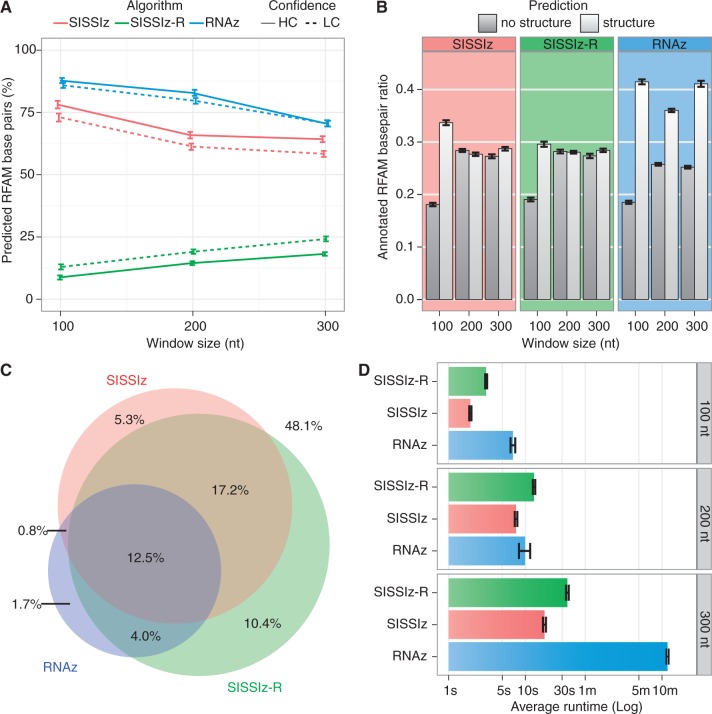
Comparative performance of consensus-based ECS algorithms. (**A**) Proportion of correctly predicted RFAM base pairs for each algorithm in function of the window size, for high-confidence predictions (RNAz probability ≥ 0.9, SISSIz Z-score ≤ −4). Error bars indicate the 95% confidence interval. (**B**) Arithmetic mean of the ratio of annotated RFAM base pairs in subalignments harboring high-confidence predictions versus that which fail to produce high-confidence predictions. Error bars indicate the standard error. (**C**) Relative exclusive overlap between RNAz and both variants of SISSIz. Values represent the percentage of total sampled alignments that produce high-confidence predictions by either algorithm. (**D**) Average runtimes per sample of the different tested algorithms in function of the sampled alignment length on a 2.66 GHz processor. Error bars represent the standard error.

We next investigated the topological quality of predicted RNA secondary structures, as the underlying consensus structure generation tool (RNAalifold) is highly dependent on the quality of the input alignment. Both tested algorithms were expected to perform similarly given their common usage of RNAalifold, yet RNAz high-confidence predictions (RNA SVM-class probability ≥ 0.90) recover slightly more annotated RFAM base pairs than SISSIz (Figure 2A). This suggests that the topological quality of ECS predictions by RNAz predictions is more accurate than those from SISSIz, which highlights the strength of RNAz’s SVM classifier. It also raises the possibility that RNAz predictions are biased toward its SVM’s training data, which is less likely for SISSIz predictions given its unsupervised nature. Indeed, the specific use of the Structural Conservation Index (SCI) by RNAz favors predictions that contain a higher density of base pairs, whereas SISSIz predictions are less affected by this parameter (Figure 2B). In combination to its relatively weaker sensitivity, these results suggest that RNAz’s SVM could be refined for genome-wide sliding-window scans by including a more diverse set of positive controls, such as the data used for this study. Despite the higher sensitivity of the RIBOSUM-enabled version of SISSIz, its predictions do not reproduce validated structural topologies as accurately as the other algorithms. Consistent with its higher sensitivity, SISSIz with RIBOSUM predictions encompass >85% of SISSIz’s or RNAz’s predictions (Figure 2C), which further substantiates its use for genomic screens. The majority (91%) of high-confidence RNAz predictions are also detected by either variant of SISSIz, whereas RNAz predictions overlap just over 37% of predictions from either version of SISSIz.

In sliding-window screens, precision is compromised for speed by limiting the sampled alignments to a fixed size. The impact of the sampled alignment size (i.e. window) was also tested through our tailor-made benchmarking pipeline. Our results suggest that the length of alignments has more impact than alignment depth—i.e. number of sequences—for both scoring variants of SISSIz, yet less so for RNAz (Figure 1). This confirms that longer windows are preferable to smaller ones in a genome-wide screen, as they are likely to encompass more ribonucleotide base pairings. Nonetheless, >2×10^7^ predictions would be performed in a genome-wide screen on standard multiple genomic alignment data using a window size of 300 bases that overlap by 50%. The difference in average speed between all algorithms favors SISSIz, surprisingly, as RNAz is promoted as one of the faster algorithms for genome-wide screens. In its current release, RNAz struggles with longer alignments as it undergoes full SVM training when the parameters of the input data fall outside the inherent classifier’s range (Figure 2D). Overall, these results highlight SISSIz’s strength at detecting evolutionary constrain on higher-order structures, while supporting the use of RNAz for more conserved sequence alignments. The reported sensitivities can be viewed as a theoretical maximal performance measure of sliding-window approaches for detecting conserved RNA structures.

Multiple genome alignments lack the refinement and precision of RFAM-sourced structural alignments, as their underlying heuristics tend to minimize gap content to maximize sequence homology. Sequence-based alignment leads to diminished frequency of consecutive nucleotides in the alignment consensus, which significantly impacts base stacking energy contributions in minimum free energy folding of RNA sequences (23). We submitted RFAM alignments to a sequence-based alignment tool to measure the extent at which structural information is dispersed during the construction of genomic alignments, and how this consequently affects consensus structure prediction (see ‘Materials and Methods’ section). Unsurprisingly, the measured sensitivities drop overall in this emulated genomic alignment benchmark. RNAz maintains roughly the same prediction sensitivities compared with the RFAM-sourced structural alignments (Table 1, Supplementary Figure S4), while both variants of SISSIz present weaker performance on the emulated genomic alignments for most MPI ranges, with the notable exception of alignments presenting very weak conservation values (<50%). The disruption of homologous helices in the native RFAM by sequence alignment algorithms can scramble *bona fide* signatures of covariant base pair mutations, thus reducing the sensitivity of predictions. Yet when sequence conservation is faint, i.e. well within the twilight zone, minimizing gaps increases the normalized Shannon entropy that can consequently provide false covariation signals for paired bases in the consensus, which should be reflected in specificity testing (see below). Overall, the differences between prediction rates from native RFAM alignments and emulated genomic alignments suggest that classical sliding-window approaches to ECS detection suffer from high false-negative rates.

### Measuring specificity

We first measured specificity (as defined in ‘Materials and Methods’ section) by producing simulated alignments for each sampled subalignment with the SISSI algorithm, which ensures that each alignment’s sequence characteristics are conserved in the simulated null alignment. At the defined confidence thresholds, all surveyed algorithms present a false-positive rate (1-specificity) ≤1% (Table 1), a substantial improvement from the ∼50% false discovery rate of the tools used in the original ENCODE scans (18,20). These results are a direct consequence of considering dinucleotide composition in the background models and scoring metrics of consensus structure prediction.

To circumvent the potential redundancy bias of using the same null model for true-negative generation and background scoring (i.e. SISSIz predictions intrinsically uses SISSI), we also randomized alignments for specificity testing with the independent program MULTIPERM that also maintains dinucleotide composition (47). With the exception of RNAz, which predicts fewer false positives in the MULTIPERM data set, the specificity of both variants of SISSIz is lower while nonetheless remaining >90% (Table 1). The lower specificity of SISSIz predictions on MULTIPERM true negatives, particularly for very low sequence conservation scores (Supplementary Figure S5), can explain the increase in sensitivity for poorly conserved true-positives alignments that have been realigned (Supplementary Figure S4). The average MPI of the MULTIPERM alignments differs on average by 7.4% from the native RFAM alignments, versus 0.2% using SISSI (Supplementary Figure S6). This indicates that MULTIPERM is not as accurate as SISSI in reproducing the precise sequence characteristics from native alignments, therefore reducing the reliability of derived specificity values.

### Performance of ECS algorithms on genomic data

Given the apparent limitations of generating true negatives from positive controls, we also surveyed genomic data to accurately evaluate the specificity of the tested ECS prediction algorithms. The RFAM-derived alignments, used to measure maximal prediction sensitivity, present analogous RNA structures composed of sequences that are not necessarily orthologous, unlike the evolutionary sequence data from multiple genome alignments. To estimate the specificity of RNAz and SISSIz in genomic screens, we applied alternative and independent shuffling strategies to the consistency-based EPO genome alignments of 35 eutherian mammals from chromosome 10 (see ‘Materials and Methods’ section). Albeit not ideal, the use of scrambled alignments as negative controls is preferable to alignments from genomic loci suspected of being devoid of function, such as ancient repeats that are commonly used to calibrate evolutionary conservation metrics. These ‘neutrally’ evolving loci are possibly functional, which while controversial, means that one cannot be confident that any existing genomic sequence is a true negative (13).

The independent dinucleotide-controlled alignment randomization algorithm MULTIPERM was used to produce negative controls from genomic alignments, both on its own and combined to randomization of the entire syntenic alignment block with SISSI. Although SISSI’s null model reproduces the sequence characteristics of the native alignments with high fidelity, the combination of both SISSI and MULTIPERM alignment simulations avoids potential algorithm-specific biases that may be encountered when using only a single randomization strategy. The ensuing alignments provide a compromise to false-positive evaluation with reduced bias and without assumptions of nonfunctionality. Table 2 describes the impact of both strategies on the false discovery rate of the ECS prediction approach described below, highlighting the discriminative power of the used tools.

**Table 2.** Effect of randomization strategies on the calibration of genomic false discovery rates

Randomization strategy	Surveyed alignments (200 nt)	RNAz	SISSIz	SISSIz + RIBOSUM	Normalized FDR (%)
		P_0.01_	Z_0.01_	Z_0.01_	
Multiperm (window)	1.17 × 10^6^	64%	−5.0	−3.1	21.7
SISSIz (block) + Multiperm (window)	1.07 × 10^6^	26%	−2.5	−2.0	4.9

Values are for 99% specificity (*P* = probability of comprising a conserved RNA structure as calculated by RNAz’s SVM RNA class probability metric; Z = Z-score from normal distribution).

**Figure 3.**
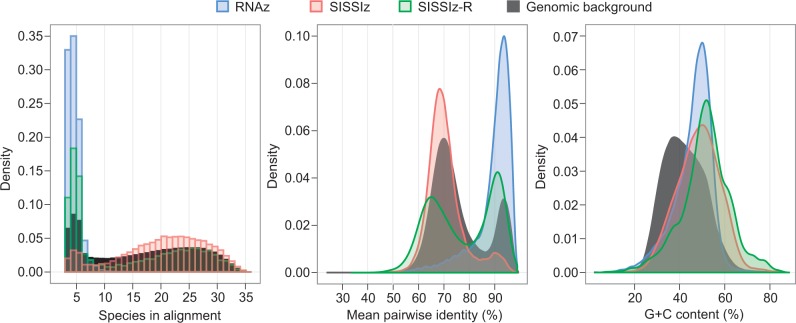
Sequence characteristics of high-confidence predictions for human chromosome 10. The kernel density estimates of the distribution of sampled species, MPI and G + C content are contrasted for all queried alignments and each subset of high-confidence hits (RNAz SVM RNA-class probability ≥ 90%; SISSIz Z-score ≤ −4). The genomic background consists of all sampled alignments.

We next investigated the performance of RNAz and SISSIz on native EPO genomic alignments. EPO alignments are more robust than alignments produced with TBA/Multiz, which have been used in previous structural characterization endeavours. They present substantially longer syntenic blocks and are >500 times less fragmented than Multiz alignments (49,50), which translate to better sampling coverage in sliding-window screens. Using a sliding-window strategy (see ‘Materials and Methods’ section), we compared the distribution of alignment characteristics for predicted ECSs with the genomic background to evaluate how both tools perform on genomic data (Figure 3). RNAz predictions are enriched for alignments with less than nine species or those presenting >80% mean pairwise sequence identity, which mainly consist of primate specific lineages (not shown). Conversely, SISSIz predictions are synonymous with alignments containing less conserved sequences that evade detection by RNAz, yet compose the bulk of alignments with >10 species. The lack of hits with higher MPI values highlights SISSIz’s difficulty to create a phylogenetic model when confronted with little sequence variation. Similarly, SISSIz is prone to generating excessively strong scores when its background distribution has a low average minimum free energy score or small variance (Supplementary Figure S1). The RIBOSUM variant of SISSIz overcomes these caveats as the distribution of sequence characteristics associated to high-confidence predictions is comparable with the genomic background, although this comes at the cost of an average runtime twice that of SISSIz (Figure 2D). Because these methods all have their peculiarities, selecting the optimal program given the sequence characteristics of an input alignment would thus be advantageous for genome-wide screens, where computational complexity is an eminent limiting factor.

### Optimized genome-wide detection of mammalian ECS

Based on the above-mentioned benchmarking results, we constructed a hybrid algorithm for large-scale comparative genomic elucidation of RNA secondary structure conservation throughout mammalian evolution, using consensus sequence-based prediction tools. Our hybrid algorithm processes genomic alignments to remove extraneous or deleterious information (such as identical sequences or high gap content) and submits the resulting overlapping windows to the optimal structure prediction tool given the alignment’s sequence characteristics (see ‘Materials and Methods’ section and Supplementary Figure S2). When applied to the EPO alignments of 35 eutherian mammals, the process completes in just over 130 000 CPU h (∼15 years) and yields >4 million alignment windows predicted to contain ECSs (Supplementary Data S4). The bulk of the predictions from the hybrid algorithm were sourced from SISSIz (30% with RIBOSUM scoring; 43% without), while RNAz was used for the remaining 27%. Because our benchmarking was performed on MAFFT-GINSI alignments, we implemented this software in our analytic pipeline to enable direct comparison between the results of the genomic screen and those from the RFAM benchmarking. Based on tests from chromosome 10, the additional realignment using MAFFT was beneficial in that ∼10% more high-confidence ECS predictions were generated than when using the native EPO alignments (not shown). However, this improved performance comes with an ∼50% increase in the computational runtime. Given that EPO alignments generate less ECS predictions than MAFFT-derived predictions, we consider that the native EPO alignments are sufficient to estimate a lower bound on the prevalence of ECSs in mammalian species.

**Figure 4.**
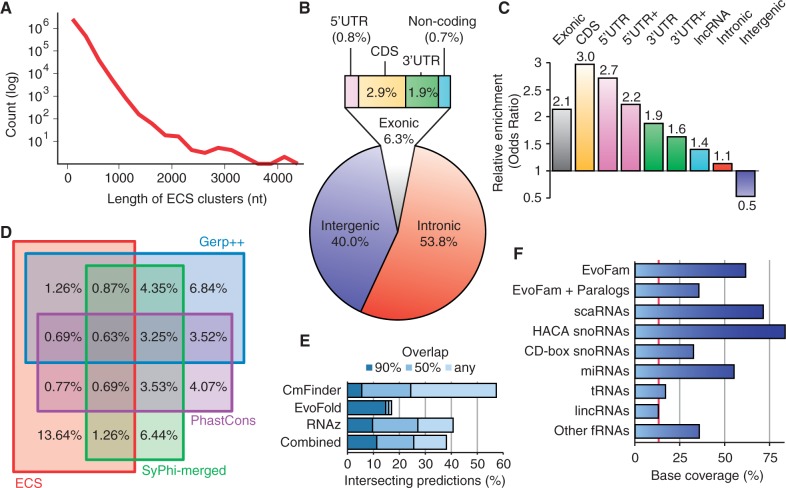
Genomic coverage and distribution of ECS predictions. (**A**) Size distribution of predicted ECS clusters. (**B**) Genomic distribution of ECS predictions with respect to the comprehensive GENCODE (version 14) genome annotations (51). Intergenic regions are defined as nonintronic or exonic regions. (**C**) Enrichment of ECS predictions in specific genomic features. The odds ratios are calculated as the ratio of ECS:nonECS base coverage in the specified genomic features compared with that outside said features, as defined by GENCODE annotations (version 14). UTR+ regions correspond to annotated untranslated regions with 250 and 2500 additional nucleotides flanking the 5′- and 3′-ends, respectively. (**D**) Venn diagram of the inclusive overlap between ECS predictions and known sequence-constrained elements. SiPhy-merged corresponds to the combined SiPhy-ω and SiPhy-π sets from (52). Mammalian PhastCons elements were extracted from the UCSC genome browser (hg19). GERP++ elements for 35 eutherian mammals were downloaded from Ensembl (release 65). Both SiPhy and PhastCons elements are derived from Multiz alignments, whereas ECS and Gerp++ are derived from EPO alignments of 35 eutherian mammals. (**E**) Fraction of predictions from previous screens that partially overlap the ECSs from SISSIz and RNAz disclosed in this study. CMfinder (version 0.2)-predicted RNA structures are taken from the ENCODE pilot project data (18), which surveyed Multiz alignments of 16 vertebrates. EvoFold (version 2.0) predictions stem from the mammalian portion of Multiz 41 vertebrate alignments (53). RNAz (version 1.0) predictions stem from Multiz alignments of eight vertebrates as reported in (19). Intersections were performed with bedTools (54). (**F**) Detection of known and putative functional RNAs: microRNAs from miRBase 15 (55); small nucleolar RNAs and small Cajal body-specfic RNAs from snoRNABase 3 (56); transfer RNAs from tRNAscan-SE 1.23 (57); large intergenic ncRNAs from the Human Body Map (58); EvoFam ECS predictions (and paralogs) from 29 mammals (53); other RNAs corresponding to a comprehensive set of structural RNA annotations (http://moma.ki.au.dk/prj/mammals/). The red line indicates the observed genomic background coverage (13.6%) by ECSs reported in this manuscript.

The resulting ECS predictions encompass 18.5% of the surveyed alignments that, in turn, span across 84.1% of the human genome (GRCh37 assembly), suggesting that a substantially greater proportion of mammalian genomes is conserved at the level of RNA structure than previously thought. Furthermore, our optimized prediction pipeline entails an unprecedented false discovery rate between 4.7 and 21.7% (sensitivity/1-specificity), depending on the source of negative controls (Table 2). To exclude the possibility that short consensus structures spanning only a fraction of the sampled alignment might inflate predictions, the genomic coordinates were truncated to exclude regions flanking the outermost paired bases from the predicted consensus structure, yielding a total genomic coverage of 13.6% (Supplementary Data S5). This includes 116 657 clusters of three or more intersecting predictions, a figure that doubles (224 475) when including hits falling within 100 nt of any ECS boundary in the same orientation of transcription (Figure 4A).

With regards to the genomic distribution of hits, the majority of predictions lie within intronic and intergenic regions as annotated in GENCODE version 14 (51) (Figure 4B). Predictions are roughly 2-fold enriched (odds ratio) in annotated exons, with the highest enrichment observed in protein-coding regions (Figure 4C). This is consistent with experimental data from genome-wide RNA structure profiling in yeast (59) and alternative computational approaches (60–62). In addition to the observed enrichment for ECS predictions in annotated and extended mRNA untranslated regions, we measured the distribution of cumulative distances between observed ECS predictions and the extremity of the nearest coding sequence. The analysis reveals that there is a relationship between the position of ECS predictions and protein-coding sequences, where ECS predictions are preferentially located up to 50 kb in either direction of protein-coding genes (Supplementary Figure S7), exposing their potential involvement as *cis*-regulatory elements. Long non–protein-coding transcripts, as defined in the comprehensive GENCODE annotations, are also enriched for evolutionarily conserved RNA secondary structures but to a lesser extent than other exonic sequences. Interestingly, repeat elements display significant enrichment for ECSs as well (1.34 odds ratio, Supplementary Table S2). In fact, about half of the ECS predictions we report overlap repeat elements, although their distribution is not uniform across the different repeat families (Supplementary Figure S8 and Supplementary Table S2). The prevalence of ECS structures is strongly enriched in Alu elements, which are known to form conserved RNA secondary structures (63), as well as endogenous retrovirus repeats. Conversely, ECSs predictions are ∼2-fold under-represented (0.6 odds ratio) in the most abundant class of genomic repeats—long interspersed nuclear elements (LINEs).

The predicted ECSs we report intersect with ∼40% of all conserved RNA secondary structures reported in previous genome-wide screens (Figure 4E), which all derive from the more fragmented TBA/Multiz genome alignments. Our approach recovers >15% of EvoFold predictions (53), which are nearly always completely covered by our predictions. Conversely, we recover >50% of CmFinder annotations (18) yet their coordinates are not precisely resolved. To further validate the accuracy of the divulged predictions, we measured the overlap between ECS hits and both known and putative functional RNAs (Figure 4F). Smaller ncRNAs, such as miRNAs and HaCa snoRNAs, are well accounted for in the predictions, just as the recently published EvoFam predictions from 29 mammals (52,53). Annotated ncRNAs that are less well detected include the largely unstructured CD-box snoRNAs and tRNAs, of which a majority source from alignments that were not sampled by our methodology. Indeed, 311 of the 625 annotated tRNAs and pseudo-tRNAs in the human genome are present in alignments longer than 200 nt that contain less than three nonidentical sequences (64), thus would not be sampled in the current implementation of this genome-wide screen. Our ECS predictions span across 38.4% of all nucleotides associated to the latter tRNAs. The low recall of tRNAs is thus a consequence of the rapid turnover of tRNA genes at individual loci (65). Nonetheless, the bulk of our predictions fall outside annotated transcripts and within introns, providing new evidence that many functional elements remain to be characterized in mammalian genomes.

We next questioned what proportion of the predicted ECSs constitute novel constrained regions by measuring overlap with known sequence-constrained elements, which are currently estimated to compose between 4.6 and 10% of the human genome (52,66). For placental mammals, the combined (high-confidence) data from these reports encompass 9.2% of the human genome, whereas the majority (87.8%) of the ECS predictions reported herein lie outside annotated sequence-constrained elements (Figure 4D). We investigated whether this dichotomy was a consequence of ECS predictions derived from primate-specific lineages, which display higher than average sequence homology compared to deeper alignments. Primate-specific lineages may inflate the proportion of ECS predictions that do not overlap with sequence-constrained elements as they are common in the 35-way EPO multiple genome alignments, as represented by the narrower, outer-most peak of the bimodal distributions of the MPI and the number of species in Figure 3. In our pipeline, RNAz is optimally employed to predict conserved RNA structures within these higher ranges of sequence conservation. When removing RNAz-derived predictions from our data set, the remaining ECS predictions encompass 8.9% of the genome and overlap only 18.1% of annotated sequence-constrained elements (versus 13.6% and 12.2% with RNAz, respectively).

We also compared the sequence composition distribution of ECS-associated alignments between known sequence-constrained elements and novel structure-constrained predictions to further emphasize the structural nature of our findings and to accentuate the divergence between ECS predictions and sequence-constrained elements (Supplementary Figure S9). Indeed, the distribution of MPI values for ECS predictions contrasts and complements that of sequence-constrained elements. The predictions that do not overlap with sequence-constrained elements are produced from alignments with lower sequence homology for all three employed algorithms. The dichotomy between the genomic loci we expose as being under negative selection at the RNA secondary structure level and those reported to be under sequence constraint reinforce the novelty of our findings, while suggesting that this is not due to the consideration of primate-specific lineages in our data. Taken together, these results substantiate the prevalence of higher-order structure complexity in mammalian genomes and suggest that the extent of purifying selection in mammalian genomes has been hitherto underreported.

### Structural congruence in human

The nature of consensus-based structure predictions does not guarantee that any given sequence in the queried alignment will form an RNA secondary structure compatible with that of the consensus. The RNAalifold algorithm, inherent to both algorithms implemented in our pipeline, only requires that half of all the input sequences form a consensus. Therefore, the human sequence may not be compatible with the consensus secondary structure that stems from a primary structure alignment. To increase the reliability of the reported predictions, we measured the likelihood that a human RNA secondary structure is compatible with that of the consensus by comparing the structural propensity of the unconstrained, native reference (human) sequence to the same sequence that is constrained to fold into the predicted consensus structure; a metric we term structural congruence (see ‘Materials and Methods’ section for details). This process retained 1 941 247 of the predicted ECSs encompassing 6.9% of the human genome, 95.1% of which do not intersect known sequence-constrained elements (Supplementary Data S6).

Another potential limitation of the method we describe is the sampling of evolutionary shallow alignments presenting restricted sequence diversity that, by default, are structurally congruent in human. Although our pipeline implements software and preprocessing steps that reduce the impact of shallow phylogenetic sampling, limited diversity can potentially reduce the statistical power of our approach as it is premised on the identification of genetic variation that is consistent with higher-order structural conservation of RNA. For example, ∼8% (322,125) of the all ECS predictions we report are sourced from alignments displaying MPI standard deviation and normalized Shannon entropy values <2.5 and <0.05, respectively, indicative of weak sequence diversity. In such instances, RNAz is preferentially used to make ECS predictions given its use of the SCI metric as an additional source of information. Including these predictions in our study is substantiated by the relative strength of RNAz in these ranges of sequence variation, which is supported by our benchmarking results and our evaluation of the experimental false discovery rate. However, to evaluate the nature and abundance of ECS predictions derived from alignments with optimal evolutionary information, we removed predictions with ≤10 sequences from our analysis. This essentially eliminates all primate-specific alignments, while 77.4% of the resulting 688 861 predicted ECSs nonetheless fail to overlap any locus annotated as evolutionarily constrained at the level of sequence. Thus, through empirical analysis of thermodynamic stability and evolutionary patterns of base pair covariation, combined to previous reports thoroughly investigating sequence-constrained elements, we can postulate a revised lower bound of functional sequence in the human genome at ∼15.6%.

### Structural annotation of a long ncRNA

**Figure 5.**
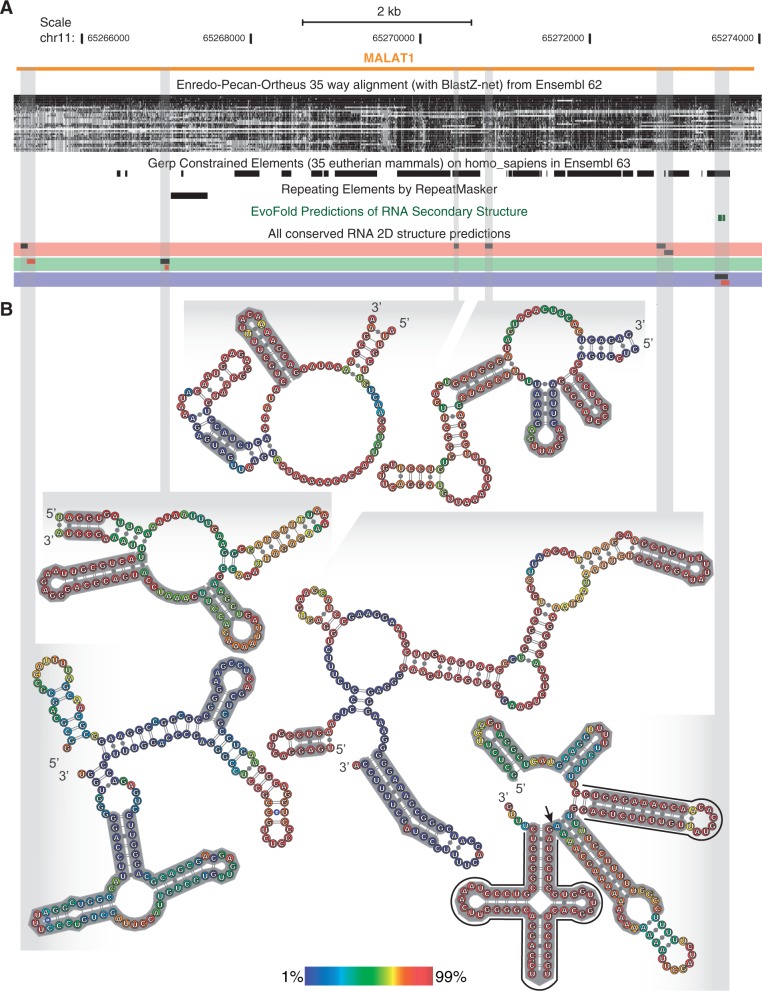
Structural characterization of the long ncRNA *MALAT1*. (**A**) UCSC genome browser (hg19) screenshot of the *MALAT1* locus with the following tracks: EPO multiple genome alignment (used to emit predictions), GERP++ constrained sequence element track, repeat elements, EvoFold evolutionarily conserved RNA secondary structure predictions, and the ECS predictions reported herein, with colors representing the algorithm used to make the prediction (SISSIz in red, SISSIz with RIBOSUM in green, RNAz in blue). Red rectangles represent ECS predictions that are structurally congruent with the reference sequence. (**B**) Human RNA secondary structures associated to predictions. Consensus RNA secondary structures were extracted from the associated alignments and used as a constraint for folding the human sequence with RNAfold (76). The base colors represent the (unconstrained) partition function base-pairing probabilities associated to the represented structures. Gray structure annotations correspond to RNAalifold consensus structures supported by conserved or compensatory mutations. The substructures outlined in black (bottom right) correspond to the previously characterized mascRNA and associated stem-loop, which are required for efficient RNAse P cleavage (cleavage site indicated with an arrow) (74). Structure representations were created with VARNA (77).

As an example of how our results can contribute to the structure–function annotation of ncRNAs, we annotated ECS predictions associated to the long (intergenic) ncRNA *MALAT1*. *MALAT1* (Metastasis-Associated Lung Adenocarcinoma Transcript 1, also known as Neat2) is an ∼7 kb unspliced ncRNA involved in regulating alternative splicing and is implicated in various cancers (67–72). It harbors an evolutionarily conserved hairpin and tRNA-like structure at its 3′-end, which is specifically cleaved by RNAse P to produce a mature polyadenylated transcript and a small cytoplasmic RNA (termed mascRNA) (73). Our high-confidence ECS predictions accurately detect the mascRNA and the conserved hairpin upstream of it (Figure 5), while identifying another conserved helical structure between the latter that was recently shown to form a stable triple helix at the 3′-end of *MALAT1* (74). Our approach also identified this additional helix at the 3′-end of the Neat1 lncRNA (not shown), which forms similar hairpin- and tRNA-like structures (75). For sake of comparison, the EvoFold algorithm only detects a substructure of the mascRNA. Furthermore, we predict several other RNA structures with evidence of evolutionary constraint in the *MALAT1* locus, the majority of which are supported by strong base-pairing probabilities in human. These results indicate that there are a variety of concise ECS motifs that can serve as putative ligands for RNA-binding proteins or for ribonucleoprotein complex formation, in *MALAT1* and (almost) every other transcribed region of the genome.

## DISCUSSION

The findings presented herein provide novel evidence for widespread functionality acting through RNA secondary structure, under the premise that negative evolutionary selection is a bona fide indicator of molecular function, in conjunction with the fact that the majority of the human genome is transcribed. Our findings provide an additional layer of support for previous reports advancing that >20% of the human genome is subjected to evolutionary selection (13,78), while suggesting that additional evidence for function can be uncovered through careful investigation of analytically involute higher-order RNA structures. Furthermore, our approach entails an empirically determined false discovery rate well below that reported in previous endeavors (i.e. 5–22% versus 50–70%) (18,20), supporting the widespread involvement of RNA secondary structure in mammalian evolution.

Our results are consistent with annotations from previous ECS screens (Figure 4E), as their intersection is of comparable magnitude when specificity is taken into consideration. The independent alignment sources, algorithm properties, preprocessing steps and methodologies used in these analyses also explain why the overlap is not greater. For example, EvoFold predictions are skewed toward lower GC content and highly conserved sequence alignments. Our method attempts to avoid the latter by removing identical sequences to optimize information content on the premise that strong sequence conservation over an adequate evolutionary distance is sufficient evidence for negative evolutionary selection. Set apart from its higher false-positive rate, EvoFold predictions nonetheless complement the ones reported in this manuscript, which present slightly higher GC content than the genomic background (Figure 3); this is a consequence of thermodynamics-based RNA structure prediction (e.g. RNAalifold) where GC base pairs contribute more to the free energy score. However, higher GC content is also associated with longer transcript half-lives in stability assays (79), thus providing additional, albeit unspecific, functional evidence to the computational predictions we report.

The expanding compendium of experimentally verified RNA structures has facilitated the benchmarking of functional RNA prediction algorithms (37,38), yet applying it to quantify structured RNAs in comparative genomic screens is not straightforward. In this work, the practicality of RFAM alignments with regards to consensus sequence-based RNA structure prediction is 2-fold: (i) to calculate an upper limit for sliding-window predictions on validated data, and (ii) to estimate the experimental error incurred by multiple sequence alignment heuristics. By comparing both results, it is possible to extrapolate the approximate accuracy of a classical scans for evolutionarily conserved RNA secondary structure.

Hence, the RNA structure predictions we report using conservative thresholds are likely to span >13.6% of the human genome we report. This number is probably a substantial underestimate of the true proportion given the conservative scoring thresholds employed, the neglect of pseudoknots, the liberal distance between overlapping windows and the incapacity of the sliding-window approach to detect base-pair interactions outside the fixed window length. A less conservative estimate would place this ratio somewhere above 20% from the reported sensitivities measured from native RFAM alignments and over 30% from the observed sensitivities derived from sequence-based realignment of RFAM data (Table 1, Figure 1 and Supplementary Figure S4). The accuracy of such extrapolations obviously depends on the reliability of the true-positive data sets, but also on how related the cataloged structures are. For instance, the covariance models used by INFERNAL (48) to generate structural RNA alignments (e.g. RFAM) from evolutionary disparate sequences can be more permissive to extreme sequence variability than what is observed in sequence alignments of a given phylum, potentially leading to divergent results on experimental data. By breaking down the control data in function of their sequence characteristics and by reproducing experimental conditions through sequence-based realignment of the input, we set the foundation for an optimized genome-wide investigation of RNA secondary structure conservation.

Our data complement recent findings from the ENCODE consortium, which report that 74.7% of the human genome is transcribed in multiple cell lines and that many novel unannotated genes are detected when sequencing RNA from subcellular compartments (3,9). In addition, the analysis of active chromatin marks and DNAseI hypersensitivity sites identified ∼45 000 novel transcription start sites without any associated RNA transcripts (80). These findings are consistent with data from high-resolution transcriptome enrichment methodologies, which reveal that even regions with sparse transcription from classic RNA sequencing experiments express a plethora of alternatively spliced RNAs in specific cells (2). These reports suggest that there is a diverse multitude of processed, noncoding transcripts in mammalian cells that fulfill regulatory roles. We postulate that the formation of higher-order structure motifs is important for specific interaction with other regulatory molecules, such as histone modification complexes (81–84). The evolutionary plasticity ensuing from higher-order structure motifs partially explains the lack of genome-wide evidence for pan-mammalian selection of novel RNA sequences reported by the ENCODE consortium. The latter nonetheless identifies an appreciable proportion of unconstrained, lineage-specific genomic elements that are required for organismal function through analysis of human genetic variation (9). Concordantly, we propose that the higher-order structural components of RNA serve as a flexible and modular evolutionary platform for the diversification of regulatory mechanisms guiding developmental ontologies, assisted by low penetrance of the affected alleles and by compensatory base pairing.

The ECS predictions we report fall largely outside known sequence-constrained elements, testifying to SISSIz’s predictive power when primary sequence conservation is weak. RNA structure algorithms predicated on measuring base-pair covariation events—the telltale idiosyncrasies of functional RNAs acting through higher-order structures—are impertinent when sequence conservation is high, where they are superseded by sequence-based approaches. The inclusion of RNAz in the hybrid algorithm is supported by SISSIz’s poorer performance when sequence conservation is high, as fewer mutations fail to generate sufficient statistical power required for SISSIz’s underlying phylogenetic model. Short external branch lengths (intrinsic to higher MPI ranges) generate a disproportionate amount of closely related sequences, thus increasing the influence of states at internal nodes of SISSIz’s phylogenetic model, potentially misleading consensus structure predictions when intermediate structures deviate by chance. The improved performance of SISSIz with RIBOSUM scoring for extreme MPI ranges can be attributed to the modified weighting of bonus energies derived from covariation events. These bonus energies have a higher contribution to the consensus than in the conventional SISSIz model, thereby favouring true conservation patterns by producing lower total free energy (23) and thus a stronger Z-score. For higher MPI values, it is likely that RIBOSUM scoring improves the discernment of ancestral correlations through the topology of the inherent phylogenetic tree. For lower MPI values, where consensus-based algorithms are accountable to the caveats stemming from the twilight zone of multiple sequence alignments, the explicit scoring of compensatory mutations (as quantified in RIBOSUM substitution matrices) is responsible for the exceptional performance of this variant of SISSIz. Furthermore, limitations caused by imperfect alignments are, in all likelihood, overcome via the discernment of each alignment’s local composition of nucleotide transitions and transversions, which can be used on their own to detect functional RNA structures (85). Although the topological accuracy of RIBOSUM endorsed predictions diverges from that in control alignments (Figure 2A), adjusting the weight of RNAalifold’s unfounded, covariation-derived bonus energy parameter may consequently provide more accurate structure prediction topologies.

The abundant ECS motifs uncovered in this work are the product of heuristic sequence alignments that sacrifice precision to accommodate speed and global orthology. Sequences conserved throughout evolution are typically used to anchor synteny maps and multiple genome alignments before the heuristic optimization of sequence similarity, which is sufficient to juxtapose orthologous sequences. We have shown that multiple genome alignments in the twilight zone can generate ECS predictions, yet the amount and quality of predictions can undoubtedly be improved (at the cost of significantly higher time complexity) by realigning the input alignments with sequence-based programs (42,86), with algorithms tailor-made for the purpose (87), or with full-fledged structure alignment algorithms [reviewed in (32,34,88)]. Although we have incorporated one realignment option to our pipeline (MAFFT-GINSI) that increased the prediction rate by ∼10% in test runs (not shown), we did not apply it to the genome-wide screen owing to a significant increase in time and memory constraints. Because EPO alignments generate less ECS predictions than MAFFT-derived predictions and given the fact that our benchmarking results represent a practical upper bound (at least for sensitivity), we consider that the native EPO alignments are sufficient to support the main claims of our manuscript. Higher resolution of ECS prevalence can also be achieved through use of longer windows with smaller steps, although the current implementation of RNAz may need to be retrained for this purpose. Small structural motifs are nonetheless functionally pertinent, as they are suspected of being involved in the binding of specific ligands and of guiding the formation of higher-order structures (82,89).

Although this work identifies millions of putative functional RNA structures, their exact functional nature remains largely uncharted. One way to achieve this is by clustering them into families based on their structural topologies, thus deriving function via homology—an approach that has been applied to smaller sets of RNA structures (53,90,91). Using current tools, these analyses are too computationally complex to be rendered practical on the extensive set of predictions we report.

Alternatively, recent advances in high-throughput sequencing of chemical- and enzyme-digested RNA structures (59,92,93) and the development of computational tools to analyze the results (94) open the door to a broader elucidation of the precise mechanisms involving higher-order RNA structures. Nonetheless, our findings reappraise the amount of functional sequence in mammalian genomes through comparative genomics by providing further evidence for widespread negative selection at the transcriptomic level. Bearing in mind that the vast majority of the mammalian genome is dynamically transcribed in different cells and developmental stages (2), these observations are consistent with the hypothesis that ncRNAs are prevalent molecular conveyors of regulatory plasticity in mammals. We further suggest that the approaches taken here will progressively reveal even more evidence for genome-wide functional selection on higher-order RNA structures as they are applied to denser lineage-restricted genomic comparisons that will inevitably emerge in the near future.

## SUPPLEMENTARY DATA

Supplementary Data are available at NAR Online.

## FUNDING

University of Queensland Research Scholarship (UQRS) and an International Postgraduate Research Scholarship (IPRS) from the Australian government (to M.A.S.); Genome Research project Bioinformatics Integration Network III (BIN III), a mobility fellowship awarded by Genome Research Austria (GEN-AU); scholarship for international cooperations from the University of Vienna (to T.G.); EU FP7 project QUANTOMICS (to P.F.S.); Australian National Health & Medical Research Council (NHMRC) [Australia Fellowship 631668 to J.S.M.]. Funding for open access charge: Garvan Institute of Medical Research.

*Conflict of interest statement*. None declared.

## Supplementary Material

Supplementary Data
