# Supplementary material for: Widespread purifying selection on RNA structure in mammals
Source: Nucleic Acids Res. 2013 Jul 11;41(17):8220–36. doi: 10.1093/nar/gkt596 (PMC3783177; doi:10.1093/nar/gkt596)
Supplement: Supplementary Data [file supp_41_17_8220__index.html]

Widespread purifying selection on RNA structure in mammals — Widespread purifying selection on RNA structure in mammals — Supplementary Data 

# Widespread purifying selection on RNA structure in mammals

## 

files

**Files in this Data Supplement:**

- Supplementary Data - pdf file
