# Supplementary material for: Widespread purifying selection on RNA structure in mammals
Source: Nucleic Acids Res. 2013 Jul 11;41(17):8220–36. doi: 10.1093/nar/gkt596 (PMC3783177; doi:10.1093/nar/gkt596)
Supplement: Supplementary Data [file supp_gkt596_nar-00268-r-2013-File007.pdf]

CONTENTS

FIGURES 2
Supplementary Figure 1 2
Supplementary Figure 2 3
Supplementary Figure 3 4
Supplementary Figure 4 5
Supplementary Figure 5 6
Supplementary Figure 6 7
Supplementary Figure 7 8
Supplementary Figure 8 9
Supplementary Figure 9 10

TABLES 11
Supplementary Table 1 11
Supplementary Table 2 12

DATA 13
Supplementary Data 1: 89 Full RFAM structure alignments used to generate data sets 13
Supplementary Data 2: Native RFAM sub-alignments used for benchmarking 13
Supplementary Data 3: Emulated genomic RFAM sub-alignments used for benchmarking 13
Supplementary Data 4: Genomic coordinates of all sampled windows 13
Supplementary Data 5: Genomic coordinates of ECS predictions 14
Supplementary Data 6: Genomic coordinates of human-congruous ECS predictions 14

SOFTWARE 15
Benchmarking data set generation and scoring 15
Hybrid algorithm for evolutionarily conserved structure prediction 15
Post processing and structural congruence 15
SISSIz 15
RNAz 15

REFERENCES 16

FIGURES

Supplementary Figure 1

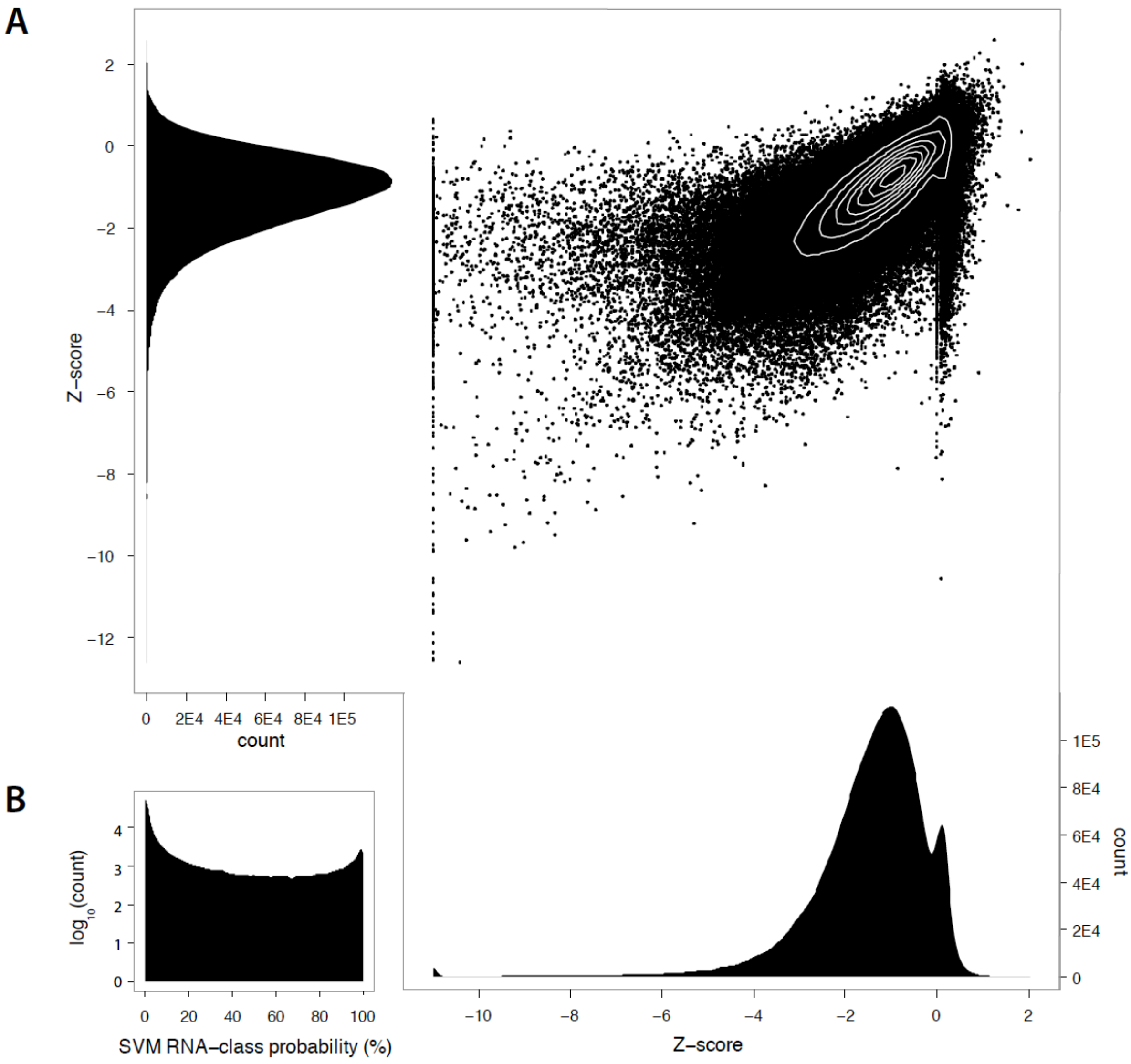

Comparative distribution of algorithm scores for chromosome 10.

(**A**) Distribution of SISSIz Z-scores (SISSIz with RIBOSUM vertical, SISSIz horizontal) and associated 2D scatter plot, where each dot represents one sampled alignment. White lines represent relative density on the Z-axis. (**B**) Log transformed distribution of RNAz scores.

Supplementary Figure 2

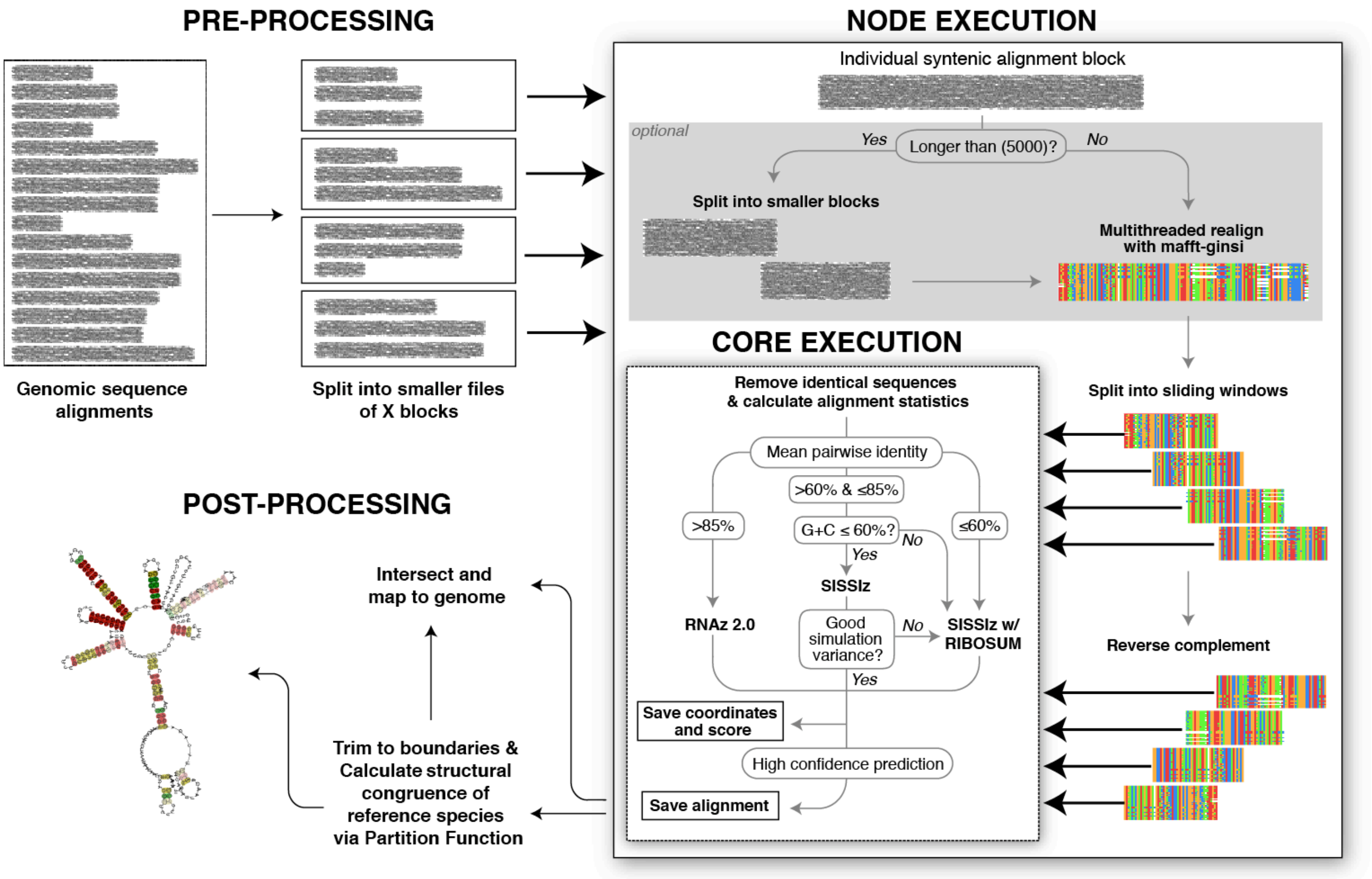

Overview of analysis pipeline and massively parallel hybrid ECS detection algorithm

Supplementary Figure 3
Length and depth of sampled RFAM data.

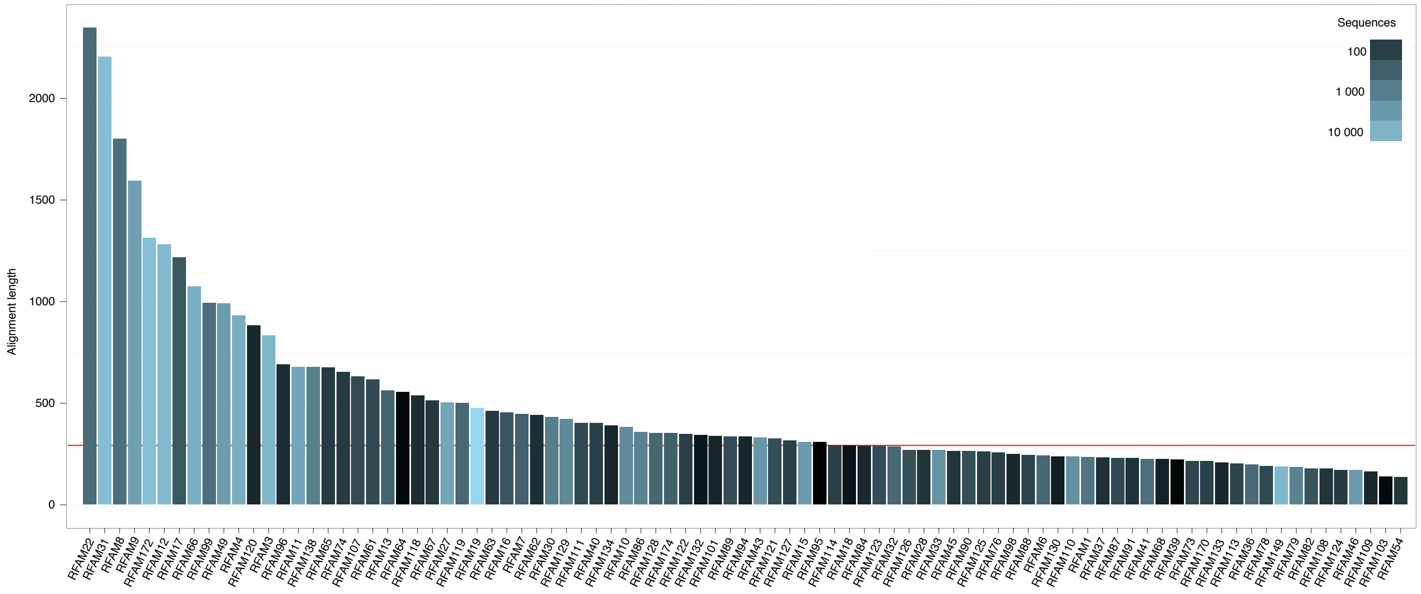

Size distribution of 89 full RFAM alignments (version 10.0) containing at least one mammalian representative. The red line indicates the inclusion threshold for the longest sampled window size (300 nucleotides) used for benchmarking the performance of consensus RNA structure prediction tools.

Supplementary Figure 4

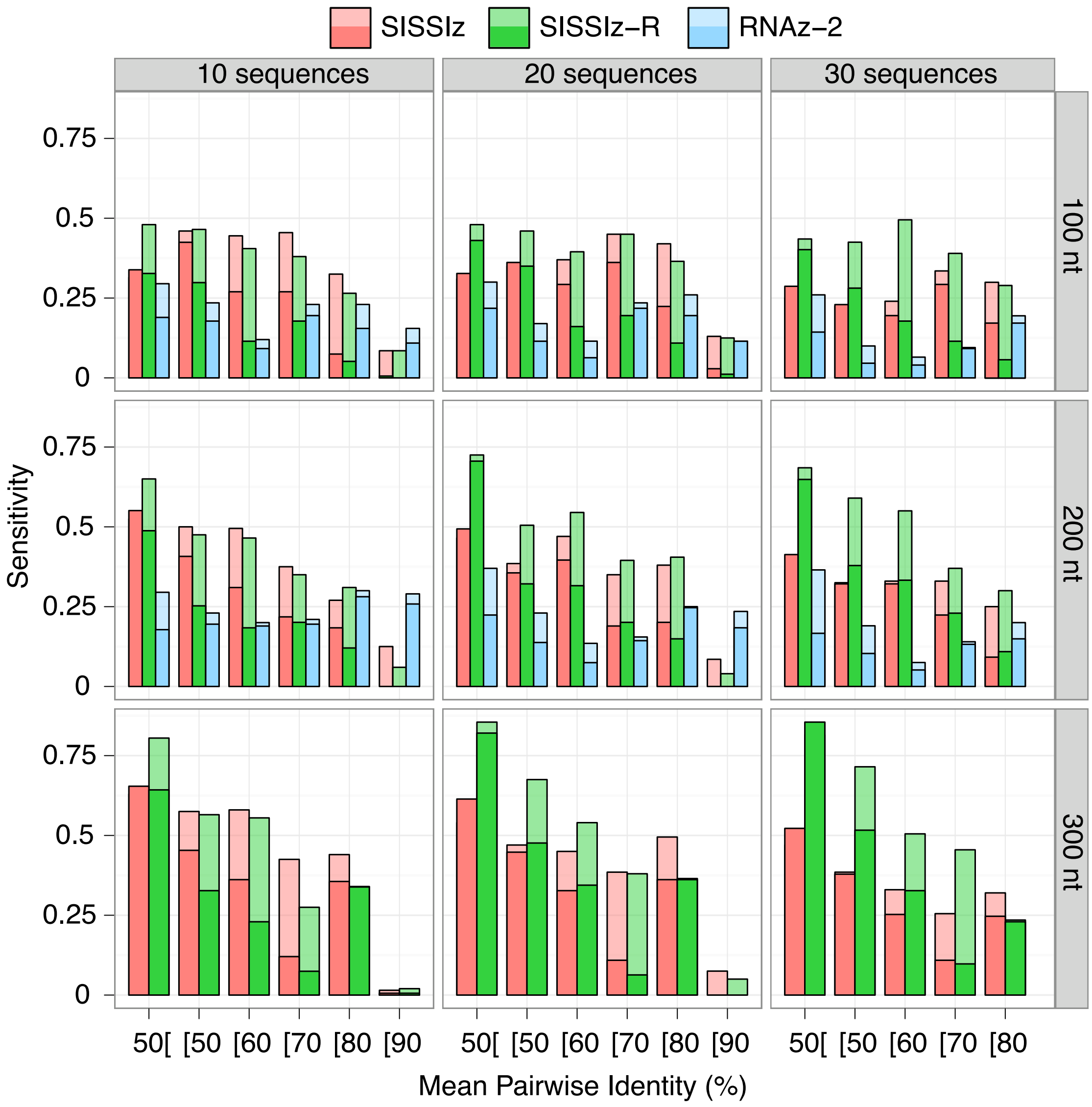

Prediction sensitivity of RNAz and SISSIz on realigned RFAM alignments.

The relative sensitivities of conserved RNA secondary structure prediction algorithms are plotted for randomly sampled partial alignments from RFAM 10.0 (Gardner et al. 2009). Opaque bars represent high-confidence predictions (RNAz probability ≥ 0.9, SISSIz P-value ≤ 0.000026) while translucent bars represent lower-confidence predictions (RNAz probability ≥ 0.9, SISSIz P-value ≤ 0.023). Each bar represents the outcome of 200 sampled alignments with RNAz version 2 (with options "-f –d –l"), SISSIz with default parameters, and SISSIz with RIBOSUM parameters (option "-j") for all indicated window sizes, sequence depths, and mean pairwise identity ranges. The latter are indicated by their lower bound values on the x-axis. Alignments were stripped of gaps and realigned with Mafft-ginsi (Katoh and Toh 2010) prior to window selection.

Supplementary Figure 5

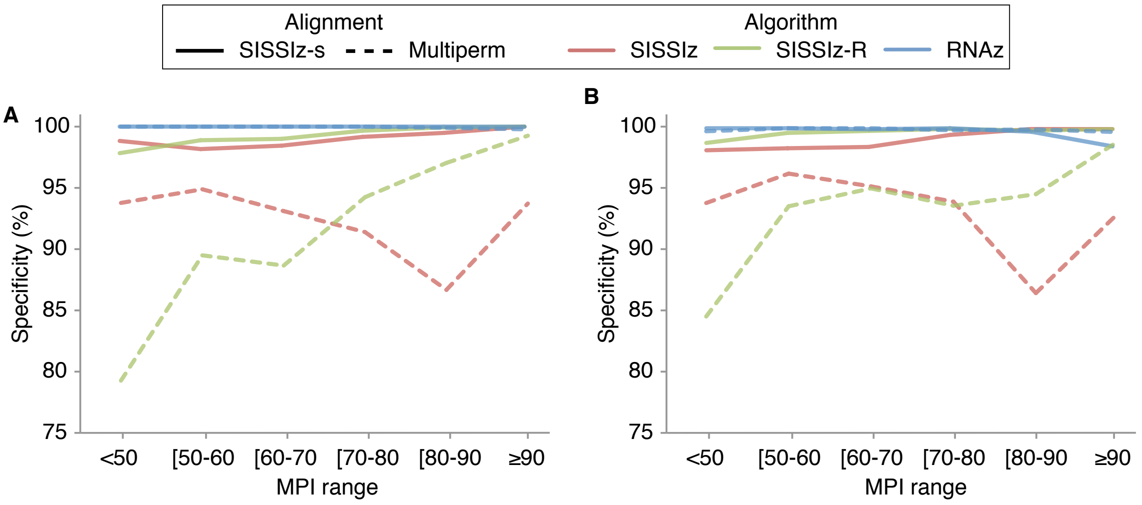

Prediction specificity in function of MPI ranges for shuffled RFAM alignments

(**A**) Native RFAM alignments; (**B**) MAFFT-derived alignments. All sub-alignments used for sensitivity testing were randomized with both SISSIz and Multiperm (Anandam et al. 2009), independently, and then scored with RNAz and both varieties of SISSIz. A fair-confidence threshold was used to discriminate false-positives and true negatives (SVM RNA-class probability ≥75% for RNAz; Z-score ≤-3 for SISSIz).

Supplementary Figure 6

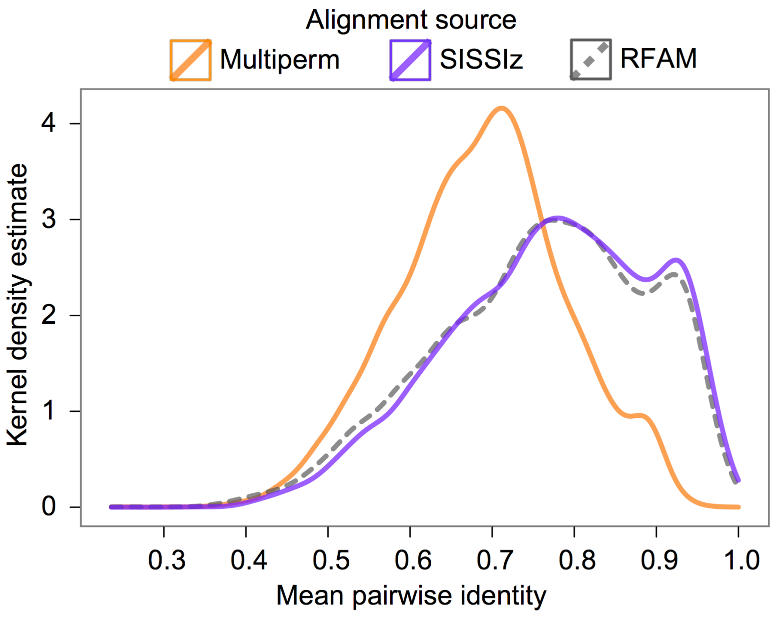

Sequence composition of alignment shuffling algorithms

Distribution of the mean pairwise identity of 10,200 sampled RFAM sub-alignments (from **Table 1**) compared to the corresponding dinucleotide-controlled randomized alignment with SISSIz using option "-s" (SISSI null model) and MULTIPERM using the default settings. The mean pairwise identity values were subsequently extracted from SISSIz's output.

Supplementary Figure 7

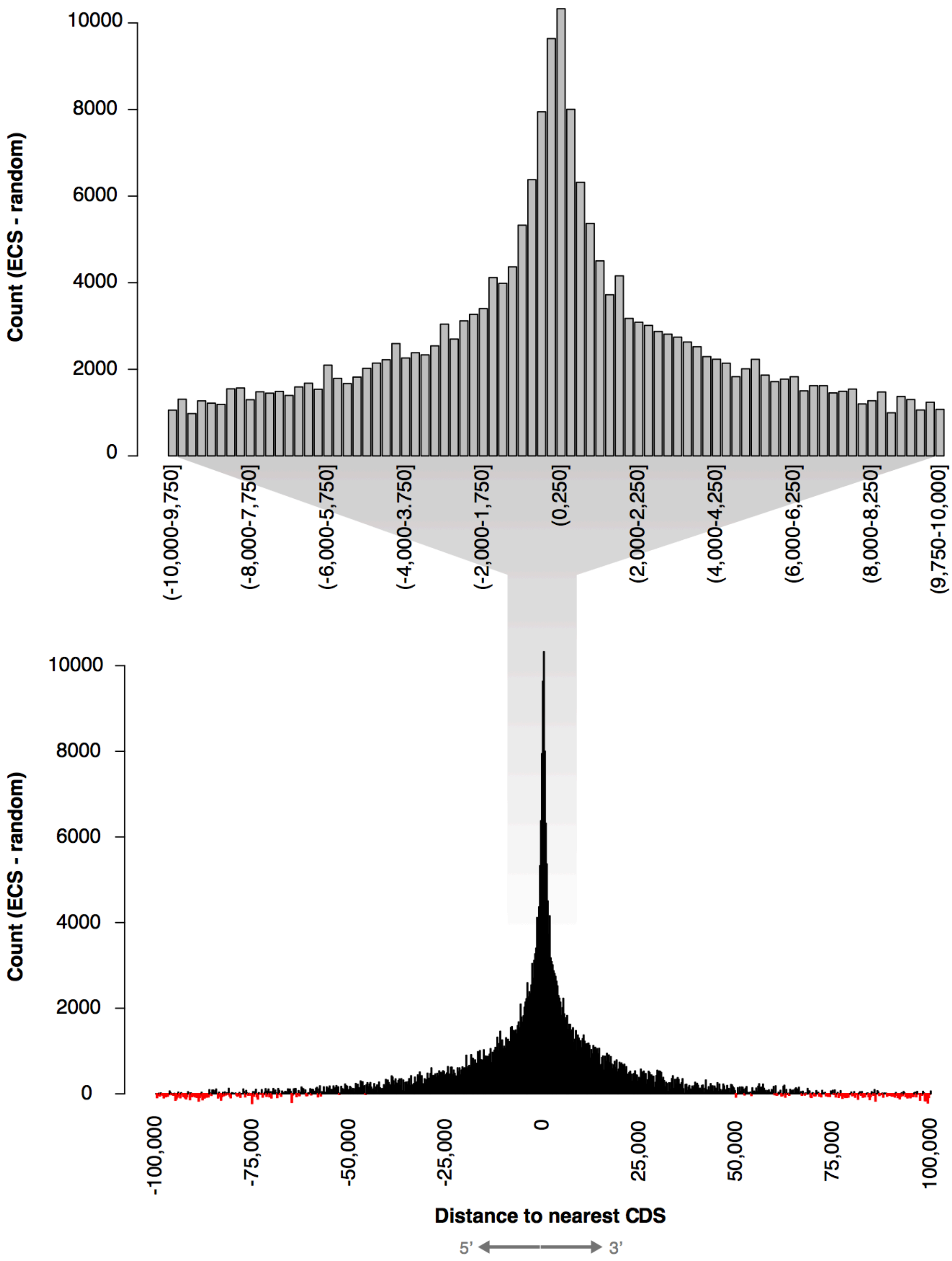

Enrichment of ECS predictions near protein coding genes
Each bar indicates the amount of ECS predictions that are located within the specified distance to the nearest protein-coding gene (CDS). The values were normalized by subtracting values obtained from equivalent coordinates that were shuffled (per chromosome) within the confines of the sampled genomic space using the BEDTOOLS suite (Quinlan and Hall 2010).

Supplementary Figure 8

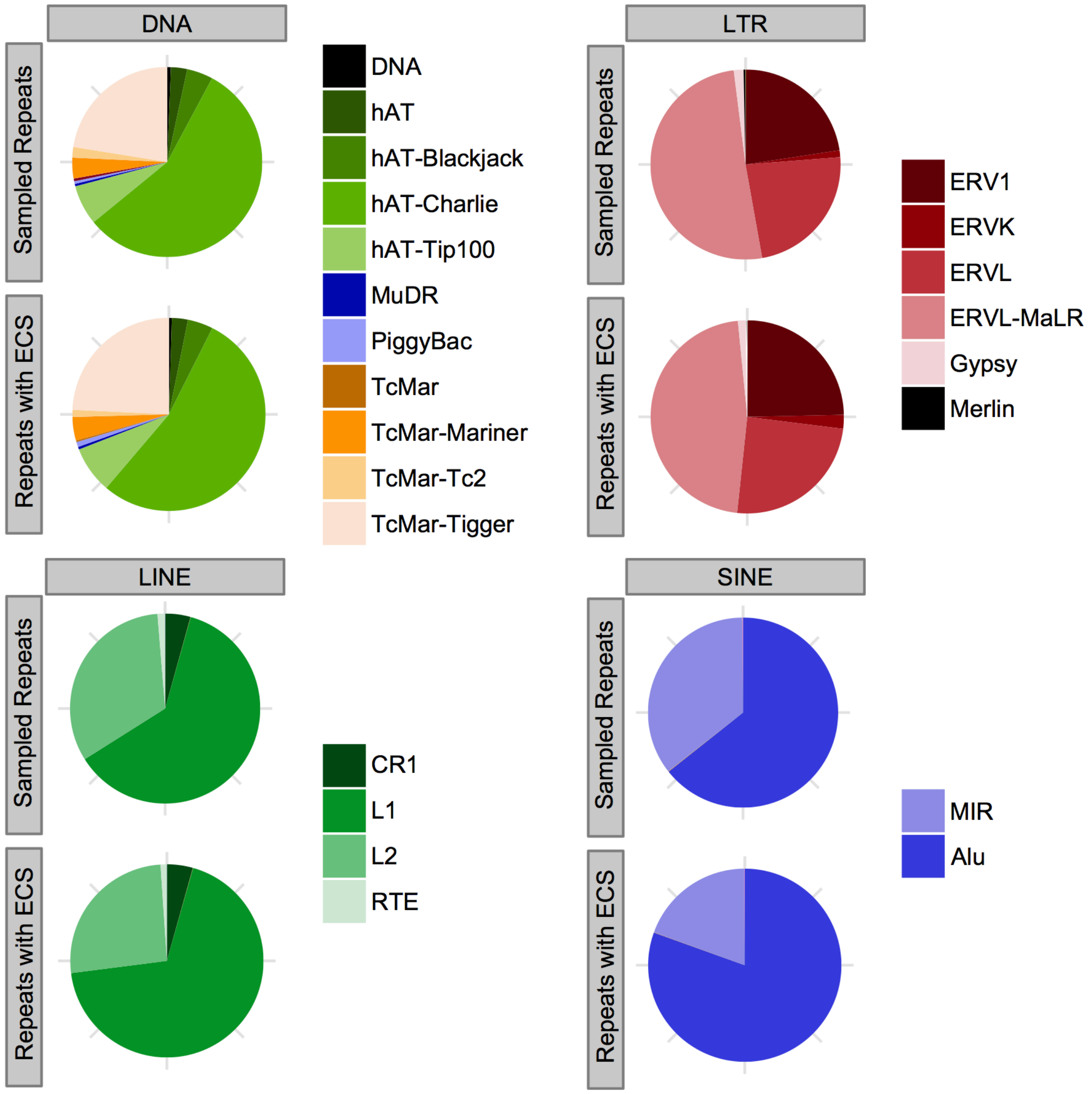

Relative composition of repeat elements
The composition of repeat element families in the 4 most abundant classes (as annotated in the RepeatMasker track from the UCSC genome browser) is contrasted between all sampled genomic coordinates (upper pie-charts) and the repeats that harbor ECS predictions (lower pie charts). DNA:DNA repeat elements; LTR:Long Terminal Repeat elements; LINE:Long Interspersed Nuclear Elements; SINE: Short Iterspersed Nuclear Elements.

Supplementary Figure 9

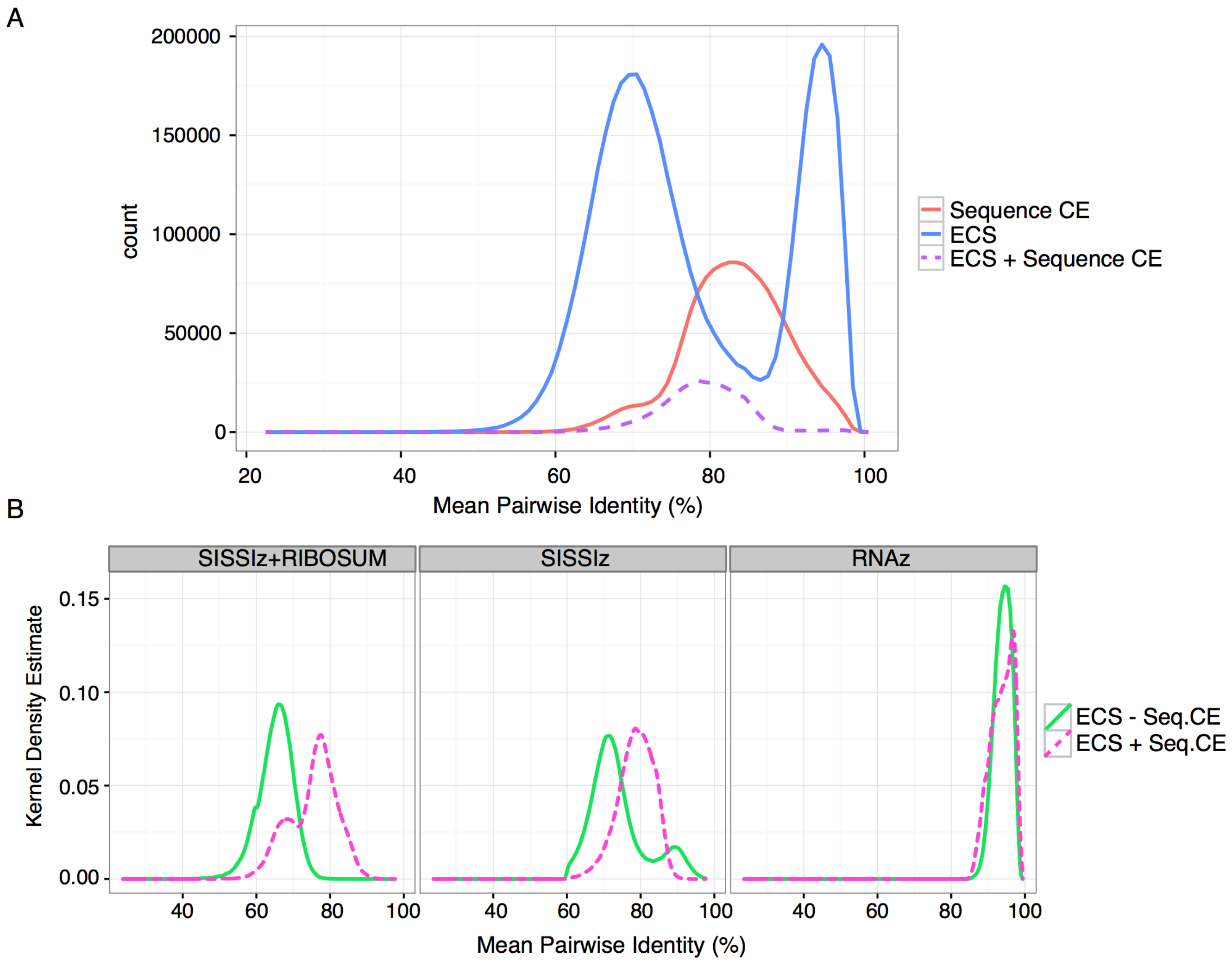

Comparative sequence similarity of constrained sequence elements and ECS predictions (**A**) Distribution of sequence similarity (mean pairwise identity) of ECS predictions and sequence constrained elements in the genomic regions sampled by our pipeline. The sequence constrained elements consist of the pooled and merged coordinates of GERP++, PhastCons and SiPhy (omega & pi data sets —converted from hg18 to the hg19 coordinates via the UCSC genome browser liftover program). The dashed line represents the fraction of sequence-constrained elements intersecting both datasets. (**B**) Comparative density estimates of the sequence composition in ECS predictions between alignments that overlap sequence-constrained elements and those that do not, in function of the algorithm employed. N.B., SISSIz with RIBOSUM scoring and RNAz predictions seldom overlap with sequence-constrained elements—the density estimates reflect the relative composition, not the relative abundance. The latter can be inferred from (**A**).

TABLES

Supplementary Table 1

Summary of RFAM full structural alignments used in this work.

RFAM ID	Description	RFAM ID	Description
**RF00001**	5S ribosomal RNA	**RF00374**	Gammaretrovirus core encapsidation signal
**RF00003**	U1 spliceosomal RNA	**RF00378**	Qrr RNA
**RF00004**	U2 spliceosomal RNA	**RF00387**	FGF-1 internal ribosome entry site (IRES)
**RF00006**	Vault RNA	**RF00391**	RtT RNA
**RF00007**	U12 minor spliceosomal RNA	**RF00422**	Small Cajal body specific RNA 24
**RF00009**	Nuclear RNase P	**RF00423**	Small Cajal body specific RNA 4
**RF00010**	Bacterial RNase P class A	**RF00424**	Small Cajal body specific RNA 16
**RF00013**	6S / SsrS RNA	**RF00426**	Small Cajal body specific RNA 15
**RF00015**	U4 spliceosomal RNA	**RF00427**	Small Cajal body specific RNA 23
**RF00017**	Eukaryotic type signal recognition particle RNA	**RF00447**	Voltage-gated potassium-channel Kv1.4 IRES
**RF00018**	CsrB/RsmB RNA family	**RF00448**	Epstein-Barr virus nuclear antigen (EBNA) IRES
**RF00020**	U5 spliceosomal RNA	**RF00449**	HIF-1 alpha IRES
**RF00022**	GcvB RNA	**RF00457**	Mnt IRES
**RF00024**	Vertebrate telomerase RNA	**RF00459**	Mason-Pfizer monkey virus packaging signal
**RF00025**	Ciliate telomerase RNA	**RF00461**	Vascular endothelial growth factor (VEGF) IRES A
**RF00026**	U6 spliceosomal RNA	**RF00463**	Apolipoprotein B (apoB) 5' UTR cis-reg. element
**RF00030**	RNase MRP	**RF00478**	Small Cajal body specific RNA 6
**RF00059**	TPP riboswitch (THI element)	**RF00483**	Insulin-like growth factor II IRES
**RF00062**	HgcC family RNA	**RF00484**	Connexin-32 internal ribosome entry site (IRES)
**RF00080**	yybP-ykoY leader	**RF00485**	Potassium channel RNA editing signal
**RF00100**	7SK RNA	**RF00487**	Connexin-43 internal ribosome entry site (IRES)
**RF00102**	VA RNA	**RF00492**	small Cajal body-specific RNA 17
**RF00106**	RNAI	**RF00495**	Hsp70 internal ribosome entry site (IRES)
**RF00113**	QUAD RNA	**RF00547**	TrkB IRES
**RF00115**	IS061 RNA	**RF00548**	U11 spliceosomal RNA
**RF00125**	IS128 RNA	**RF00549**	c-sis internal ribosome entry site (IRES)
**RF00126**	ryfA RNA	**RF00552**	rncO
**RF00140**	Alpha operon ribosome binding site	**RF00553**	Small Cajal body specific RNA 1
**RF00162**	SAM riboswitch (S box leader)	**RF00564**	Small Cajal body specific RNA 11
**RF00166**	PrrB/RsmZ RNA family	**RF00565**	Small Cajal body specific RNA 3
**RF00169**	Bacterial signal recognition particle RNA	**RF00582**	Small Cajal body specific RNA 14
**RF00174**	Cobalamin riboswitch	**RF00601**	Small Cajal body specific RNA 20
**RF00182**	Coronavirus packaging signal	**RF00602**	Small Cajal body specific RNA 21
**RF00216**	c-myc internal ribosome entry site (IRES)	**RF00618**	U4atac minor spliceosomal RNA
**RF00222**	Bag-1 internal ribosome entry site (IRES)	**RF00619**	U6atac minor spliceosomal RNA
**RF00223**	bip internal ribosome entry site (IRES)	**RF00621**	Beta-globin co-transcriptional cleavage ribozyme
**RF00224**	FGF-2 internal ribosome entry site (IRES)	**RF00629**	Pseudomonas sRNA P24
**RF00226**	n-myc internal ribosome entry site (IRES)	**RF00635**	Human accelerated region 1F
**RF00230**	T-box leader	**RF00636**	ncRNA Repressor of NFAT
**RF00231**	Small Cajal body specific RNA 13	**RF01086**	Long range pseudoknot
**RF00232**	Spi-1 (PU.1) 5' UTR regulatory element	**RF01118**	Pseudoknot of the domain G(G12) of 23S rRNA
**RF00259**	Interferon gamma 5' UTR regulatory element	**RF01387**	isrC Hfq binding RNA
**RF00261**	L-myc internal ribosome entry site (IRES)	**RF01417**	Retroviral 3'UTR stability element
**RF00286**	Small Cajal body specific RNA 8	**RF01492**	Listeria snRNA rli28
**RF00369**	sroC RNA		

Supplementary Table 2

Relative genomic coverage and enrichment of ECS predictions within repeat elements

Repeat Family		Genomic coverage of repeats (%)*	Genomic coverage of ECSs in repeats (%)*	Odds-Ratio**	Ln(OR)	Standard Error
LINE		20.496	2.18434	0.56	-0.59	0.0002
	CR1	0.414	0.04846	0.68	-0.38	0.0010
	RTE	0.141	0.01489	0.61	-0.50	0.0018
	L2	3.949	0.40901	0.59	-0.53	0.0003
	L1	16.001	1.71518	0.57	-0.55	0.0002
SINE		12.651	4.05554	2.91	1.07	0.0001
	ALU	9.420	3.54868	3.71	1.31	0.0001
	MIR	3.206	0.50177	0.96	-0.04	0.0003
LTR		8.820	1.57682	1.14	0.13	0.0002
	ERVK	0.172	0.05683	2.56	0.94	0.0010
	ERV1	2.412	0.44673	1.18	0.16	0.0003
	ERVL-MaLR	4.016	0.69948	1.09	0.09	0.0003
	ERVL	6.095	1.05312	1.08	0.08	0.0002
	Gypsy	0.145	0.02093	0.87	-0.14	0.0015
	Merlin	0.001	0.00004	0.37	-0.99	0.0340
DNA		3.595	0.61119	1.06	0.06	0.0003
All Repeat Elements		45.563	8.42789	1.34	0.29	0.0001

* Relative to the sampled genomic space (84.1% of non-"N" human bases)
** Calculated as the ratio of nucleotides encompassing ECS prediction to those not encompassing ECS predictions in the genomic feature of interest compared to that in the remainder of the sampled genome.

DATA

89 Full RFAM structure alignments used to generate data sets

http://www.martinalexandersmith.com/ECS/RFAM_mammalia.tgz (151 MB)
The first FASTA entry in all alignments corresponds to the consensus of the alignment. The second entry corresponds to the secondary structure mask, in dot-bracket format. Only families with at least one mammalian representative were downloaded from RFAM (ftp://ftp.sanger.ac.uk/pub/databases/Rfam/10.0/).

Native RFAM sub-alignments used for benchmarking

http://www.martinalexandersmith.com/ECS/benchmark_native.tgz (66 MB)
Includes native alignments used for **Figure 2** and **Table 1**, the associated shuffled alignments, and the corresponding sequence characteristics and ECS algorithm scores in a tab-delineated text file. See README.txt for more details.

Emulated genomic RFAM sub-alignments used for benchmarking

http://www.martinalexandersmith.com/ECS/benchmark_realigned.tgz (61 MB)
Includes mafft-ginsi realigned alignments used for **Supplementary Figure 2** and **Table 1**, the associated shuffled alignments, and the corresponding sequence characteristics and ECS algorithm scores in a tab-delineated text file. See README.txt for more details.

Genomic coordinates of all sampled windows

http://www.martinalexandersmith.com/ECS/all_sampled.bed.gz (654 MB)
6-field browser extensible data file comprising results from all surveyed windows, as reported in Methods. The name field (column 4) includes the following colon-delineated alignment statistics:

- Number of sequences;
- Raw mean pairwise identity;
- Mean pairwise identity (normalized to the shortest gapless sequence length, as reported in main text);
- Relative gap content;
- Standard deviation of pairwise identity;
- Normalized Shanon entropy;
- Relative GC content;
- Alignment algorithm used to produce score:
  s = SISSIz
  r = SISSIz with RIBOSUM
  z = RNAz-2

The score field (column 5) corresponds to -100x the Z-score when SISSIz is used, or 100x the SVM RNA-class probability when RNAz is employed.

Genomic coordinates of ECS predictions

http://www.martinalexandersmith.com/ECS/ECS_trimmed.bed.gz (88 MB)
Browser extensible data file containing all reported ECS predictions (trimmed to the outer-most helix). Fields 4 and 5 are the same as described above.

Genomic coordinates of human-congruous ECS predictions

http://www.martinalexandersmith.com/ECS/ECS_congruous.bed.gz (151 MB)
Browser extensible data file containing all reported ECS predictions defined as structurally congruous in Human (see **Methods** for details), with additional fields:

(4-5) As described above;
(7) Average base pairing probability of minimum free energy structure for human;
(8) Average base pairing probability of consensus-constrained human structure;
(9) Base pairing probability ratio (constrained/native);
(10) Minimum free energy (Kcal/mol) of constrained human sequence;
(11) Minimum free energy (Kcal/mol) of native human sequence;
(12) Minimum free energy ration (constrained/native);
(13) Length of prediction (nt);
(14) Dot-bracket secondary structure mask of RNAalifold consensus.

SOFTWARE

All source code available upon request: *martinalexandersmith[at]gmail[dot]com*

Benchmarking data set generation and scoring

http://www.martinalexandersmith.com/ECS/BuildRfamBenchmark.jar
Java Archive, executable with "java –jar BuildRfamBenchmark.jar" in command prompt.

Hybrid algorithm for evolutionarily conserved structure prediction

http://www.martinalexandersmith.com/ECS/MafScanCcr.jar
Java Archive executable with "java –jar MafScanCcr.jar" in command prompt. Supports multithreading. Requires installation of SISSIz and RNAz, with binaries linked in environmental PATH variable.

Post processing and structural congruence

http://www.martinalexandersmith.com/ECS/ParseAlifold.jar
Java Archive executable with "java –jar ParseAlifold.jar" in command prompt. Supports multithreading. Requires installation of Vienna RNA package version 1.8.5 (http://www.tbi.univie.ac.at/RNA/ViennaRNA-1.8.5.tar.gz) with binaries linked to PATH.

SISSIz

http://www.martinalexandersmith.com/ECS/SISSIz-0.3.tar.gz (3 MB)
SISSIz version used in this work (Gesell and Washietl 2008).

RNAz

http://www.martinalexandersmith.com/ECS/RNAz-2.0pre.tar.gz (11 MB)
RNAz version used in this work (Gruber et al. 2010).

REFERENCES

Anandam P, Torarinsson E, Ruzzo WL. 2009. Multiperm: shuffling multiple sequence alignments while approximately preserving dinucleotide frequencies. *Bioinformatics* **25**(5): 668-669.

Gardner PP, Daub J, Tate JG, Nawrocki EP, Kolbe DL, Lindgreen S, Wilkinson AC, Finn RD, Griffiths-Jones S, Eddy SR et al. 2009. Rfam: updates to the RNA families database. *Nucleic acids research* **37**(Database issue): D136-140.

Gesell T, Washietl S. 2008. Dinucleotide controlled null models for comparative RNA gene prediction. *BMC Bioinformatics* **9**: 248.

Gruber AR, Findeiss S, Washietl S, Hofacker IL, Stadler PF. 2010. Rnaz 2.0: Improved Noncoding Rna Detection. *Pac Symp Biocomput* **15**: 69-79.

Katoh K, Toh H. 2010. Parallelization of the MAFFT multiple sequence alignment program. *Bioinformatics* **26**(15): 1899-1900.

Quinlan AR, Hall IM. 2010. BEDTools: a flexible suite of utilities for comparing genomic features. *Bioinformatics* **26**(6): 841-842.
